# Chimera: enabling hierarchy based multi-objective optimization for self-driving laboratories†
†Electronic supplementary information (ESI) available. See DOI: 10.1039/c8sc02239a


**DOI:** 10.1039/c8sc02239a

**Published:** 2018-08-28

**Authors:** Florian Häse, Loïc M. Roch, Alán Aspuru-Guzik

**Affiliations:** a Department of Chemistry and Chemical Biology , Harvard University , Cambridge , Massachusetts 02138 , USA . Email: alan@aspuru.com ; Tel: +1-617-384-8188; b Department of Chemistry and Department of Computer Science , University of Toronto , Toronto , Ontario M5S3H6 , Canada; c Vector Institute for Artificial Intelligence , Toronto , Ontario M5S1M1 , Canada; d Canadian Institute for Advanced Research (CIFAR) Senior Fellow , Toronto , Ontario M5S1M1 , Canada

## Abstract

Finding the ideal conditions satisfying multiple pre-defined targets simultaneously is a challenging decision-making process, which impacts science, engineering, and economics. Additional complexity arises for tasks involving experimentation or expensive computations, as the number of evaluated conditions must be kept low. We propose Chimera as a general purpose achievement scalarizing function for multi-target optimization where evaluations are the limiting factor. Chimera combines concepts of *a priori* scalarizing with lexicographic approaches and is applicable to any set of *n* unknown objectives. Importantly, it does not require detailed prior knowledge about individual objectives. The performance of Chimera is demonstrated on several well-established analytic multi-objective benchmark sets using different single-objective optimization algorithms. We further illustrate the applicability and performance of Chimera with two practical examples: (i) the auto-calibration of a virtual robotic sampling sequence for direct-injection, and (ii) the inverse-design of a four-pigment excitonic system for an efficient energy transport. The results indicate that Chimera enables a wide class of optimization algorithms to rapidly find ideal conditions. Additionally, the presented applications highlight the interpretability of Chimera to corroborate design choices for tailoring system parameters.

## Abstract 2

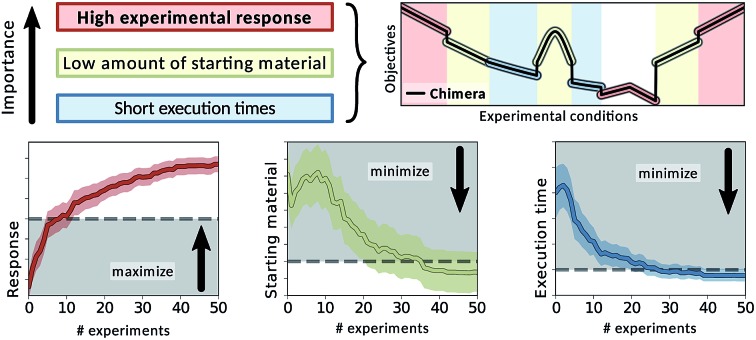
Chimera enables multi-target optimization for experimentation or expensive computations, where evaluations are the limiting factor.

## Introduction

Multi-objective optimization is ubiquitous across various fields in science, engineering and economics. It can be interpreted as a multi-target decision-making process,^1^ aiming at finding the ideal set of conditions, *e.g.* parameters of experimental procedures, theoretical models or computational frameworks, which yield the desired pre-defined targets. In chemistry and materials science, these targets can include the yield and selectivity of reactions, production cost and overall execution time of processes, or optimization of materials with properties tailored to specific needs. In general, ideal conditions for which all targets assume their desired optimal values do not exist. As a matter of fact, improving on one target might only be possible at the expense of degrading other targets.

Straightforward approaches to determine ideal conditions satisfying multiple targets are detailed systematic searches of all possible conditions. However, these approaches require numerous objective evaluations, scale exponentially with the number of conditions to be optimized, and do not guarantee locating the ideal conditions. Therefore, applications involving experimentation or expensive computations are beyond the viability of these searches as the number of conducted experiments or computations must be kept low. Thus, robust and efficient algorithms evolving on multi-dimensional surfaces are needed to identify optimal conditions within a minimum number of distinct evaluations.

These robust and efficient algorithms have the potential to open new avenues to multi-objective optimization in chemistry and materials science when combined with closed-loop experimentation as implemented in self-driving laboratories. Such laboratories combine artificial intelligence with automation, and enable the design and execution of experiments in full autonomy, without human interaction.^2^–^8^ The learning procedure suggests new conditions while accounting for the observed merit of previously conducted experiments, forming a closed-loop. Consequently, self-driving laboratories learn experimental conditions on-the-fly by continuously refining parameters to maximize the merit of the machine-proposed conditions and satisfy pre-defined targets.^9^,^10^ However, applications with multiple objectives pose the challenge of formulating an optimal solution based on tolerated trade-offs in the objectives. To address this challenge, approaches have to be capable of balancing competing criteria and identifying the conditions yielding the highest merit with respect to user-defined preferences. Herein, we propose Chimera, a versatile achievement scalarizing function (ASF) for multi-objective optimization with costly to evaluate objectives.

Recently, multi-objective optimization approaches have been successfully applied to various scenarios. Examples include the rational design of dielectric nanoantennas^11^ and plasmonic waveguides,^12^ the optimization of Stirling heat pumps,^13^ the design of thermal-energy storage systems,^14^–^16^ and optimizations on scheduling problems in combined hydro-thermo-wind power plants.^17^ However, in the aforementioned applications the merit of a set of conditions could be assessed by analytic models which were fast to evaluate computationally. As such, these optimization problems could be approached with methods identifying the entire set of solutions which cannot be further optimized in at least one of the objectives, at the expense of numerous objective evaluations. Preference information regarding specific solutions could then be expressed knowing the surface of optimal points.

In chemistry, multi-objective optimization methods have been applied to determine trade-offs in the reaction rate and yield of methylated ethers,^18^ maximize the intensity of quantum dots at a target wavelength,^19^ or balance the production rate and conversion efficiency of Paal–Knorr reactions.^20^ These optimization problems have been approached with methods that allow preference information to be expressed prior to starting the optimization procedures. As such, the optimization procedures were more efficiently targeted towards the desired goal. Preference information was provided by constructing a single merit function from all considered objectives such that the single merit-based function accounts for the provided preferences. Optimizations were then conducted on the merit-based function using single objective optimization algorithms.

The above-mentioned examples display the successful application and benefit of multi-objective optimization methods for self-optimizing reactors, illustrating how they can power self-driving laboratories. Yet, the merit-based functions employed in these examples are often handcrafted. Constructing a suitable and versatile merit-based function with little prior knowledge about the objectives is challenging.^21^,^22^ As a matter of fact, compositions of merit-based functions can sometimes require refinements after initial optimization runs as the desired preference in the objectives is not achieved.^20^

Recently, Walker *et al.* introduced a framework for formulating merit-based multi-objective optimization as constrained optimization problems for the synthesis of *o*-xylenyl adducts of buckminsterfullerene.^23^ Their approach aims to optimize a main objective, while keeping other objectives at desired levels by considering them as constraints. However, their method depends on the choice of constraints, which requires substantial prior knowledge about the objective surfaces. Therefore, the lack of a universal, general purpose method for constructing merit-based functions from multiple objectives is a challenge to design problems and appears as a major obstacle to the massive deployment of self-optimizing reactors and self-driving laboratories. Notably, we identify two main constraints: (i) objective evaluations involve timely and costly evaluations (experimentally or computationally), and thus, must be kept to a minimum, (ii) no prior knowledge is available about the surface of the objectives. In this work, we use these constraints as requirements for the formulation of Chimera.

Chimera is an approach to multi-objective optimization for experimental and computational design. It combines concepts of *a priori* scalarizing with ideas from lexicographic approaches and is made available on GitHub.^24^ Herein, we show on several well-established benchmark sets and in two practical applications how Chimera fulfills the aforementioned constraints. Our proposed method relies on preference information provided in the form of a hierarchy in the objectives. A single merit-based function is constructed from the provided hierarchy, and it shapes a surface which can be optimized by a variety of single-objective optimization algorithms. Chimera does not require detailed assumptions about the surfaces of the objective functions and it improves on the hierarchy of objectives from the beginning of the optimization procedure, without any required warm-up iterations.

This manuscript is organized as follows. We start with an overview of the multi-objective formulation, and machine-learning based algorithms. Then, we detail the implementation of Chimera, and assess its performance on multi-objective benchmark functions. Before drawing our conclusions, we further demonstrate the applicability of Chimera in an automated experimental procedure for real-time reaction monitoring, and in the inverse-design of an excitonic system for the efficient transport of excitation energy.

## Background and related work

**Fig. 1:**
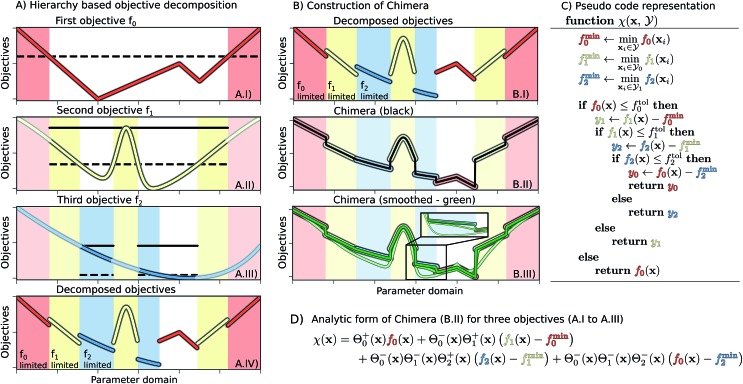
Example for the construction of Chimera from three one-dimensional objective functions. Panel (A) Illustration of the three objective functions, *f*_0_, *f*_1_ and *f*_2_, in order of the hierarchy. For constructing Chimera, each objective is considered only in the parameter region where higher-level objectives satisfy the tolerances (dashed lines). Solid lines indicate the upper objective bound in the region of interest used as a reference for the tolerance on the considered objective. The objective functions considered in different parameter regions for this example are illustrated in A.IV. Panel (B) The construction of Chimera for the considered objective. The discrete variant of Chimera (black, panel B.II) is constructed using eqn (2), which was substituted with eqn (6) to generate smooth variants (green, panel B.III) using different smoothing parameter values, where lighter traces correspond to larger parameter values. Panel (C) Pseudo code showcasing the conceptual implementation of Chimera. Panel (D) Analytic expression for the discrete Chimera variant constructed from three objective functions.

Multi-objective (Pareto) optimization is concerned with the simultaneous optimization of a set of objective functions, {*f*_*k*_}*n*–1*k*=0, where each of the objective functions, *f*_*k*_, is defined on the same compact parameter space 
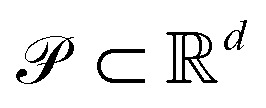
.^25^ Objectives of interest in the context of chemistry could be, for example, the yield of a reaction and its execution time. Although the desired goal of an optimization procedure is to find a point in parameter space 
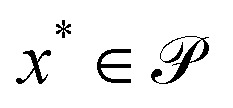
 for which each of the objectives *f*_*k*_(x^*^) assume their desired optimal value (*e.g.* minimum/maximum), objectives in multi-objective optimization problems oftentimes conflict with each other. Indeed, improving on one objective could imply an unavoidable degradation in other objectives as, for instance, shorter execution times could cause a drop in yield. As a consequence, a single global solution cannot be defined for the generic multi-objective optimization problem. This challenge is illustrated in Fig. 1A, where a set of three objective functions with global minima at different locations is presented.

### Defining and identifying solutions to multi-objective optimization problems

A commonly used criterion for determining solutions to multi-objective optimization problems is Pareto optimality.^26^ A point is called Pareto optimal *if and only if* there exists no other point such that all objectives are improved simultaneously. Therefore, deviating from a Pareto optimal point always implies a degradation in at least one of the objectives. Relating to the previous example, this corresponds to a scenario in which the execution time cannot be improved any further without a degradation of the reaction yield. As Pareto optimal points cannot be collectively improved in two or more objectives, solving a multi-objective optimization problem translates to finding Pareto optimal points. Note that for a given multi-objective optimization problem, multiple Pareto optimal points can coexist.^27^

Typically, approaches to solving multi-objective optimization problems aim to assist a decision maker in identifying the favored solution from the set of Pareto optimal solutions (Pareto front). The favored solution is determined from preference information regarding the objectives provided by the decision maker. Methods for multi-objective optimization can be divided into two major classes. *A posteriori* methods aim to discover the entire Pareto front, such that preferences regarding the objectives can be expressed knowing which objective values are achievable. This relates to knowing by how much the execution time needs to be increased to achieve a desired increase in the reaction yield. *A priori* methods instead require preference information prior to starting the optimization procedure. As such, *a priori* methods can be more specifically targeted towards the desired goal and thus reduce the necessary number of objective evaluations if reasonable preference information is provided.

*A posteriori* methods are commonly realized as mathematical programming approaches, such as Normal Boundary Intersection,^28^,^29^ Normal Constraint,^30^,^31^ or Successive Pareto Optimization,^32^ which repeat algorithms for finding Pareto optimal solutions. Another strategy consists in evolutionary algorithms such as the Non-dominated Sorting Genetic Algorithm-II,^33^ or the Sub-population Algorithm based on Novelty,^34^ where a single run of the algorithm produces a set of Pareto optimal solutions. Recently, *a posteriori* methods have also been developed following Bayesian approaches for optimization.^35^–^39^ However, determining the preferred Pareto point from the entire Pareto front requires a substantial number of objective function evaluations compared to scenarios in which only a subset of the Pareto front is of interest. Such scenarios can be found in the context of experimental design, where preferences regarding objectives like yield and execution time are available prior to the optimization procedure. As such, *a priori* methods appear to be better suited for multi-objective optimization in the context of designing experiments, as they keep the number of objective evaluations to a minimum.

A common *a priori* approach for expressing preferences for multi-objective optimization is to formulate a single cumulative function from a combination of the set of objectives which accounts for the expressed preferences (see Fig. 1B). For example, instead of considering the yield and the execution time of a reaction independently, a single objective can be constructed from a combination of simultaneous observations for the yield and the execution time. Such cumulative functions are referred to as achievement scalarizing functions (ASFs). The premise of the constructed ASF is that its optimal solution coincides with the preferred Pareto optimal solution of the multi-objective optimization problem.

Typically, ASFs are constructed with a set of parameters which account for the expressed preferences regarding the individual objectives. ASFs can be constructed *via*, for example, weighted sums or weighted products of the objectives. In such approaches, the ASF is computed by summing up each objective function *f*_*k*_ multiplied by a pre-defined weight *w*_*k*_ accounting for the user preferences. Multiple formulations of weighted sums and products exist,^40^ and methods have been developed to learn these weights adaptively.^41^ Weighted approaches are usually simple to implement, but the challenge lies in finding suitable weight vectors to yield Pareto optimal solutions. In addition, Pareto optimal solutions might not be found for non-convex objective spaces.

A second *a priori* approach consists in considering only one of the objectives for optimization while constraining the other objectives based on user preferences.^42^–^44^These approaches, referred to as ε-constraint methods, have been shown to find Pareto optimal points even on non-convex objective spaces.^27^,^45^ However, the constraint vector needs to be chosen carefully, which typically requires detailed prior knowledge about the objectives.

A third *a priori* approach, known as lexicographic methods, follows yet a different approach.^46^ Lexicographic methods require preference information expressed in terms of an importance hierarchy in the objectives (see Fig. 1A.I–III). In our example, when optimizing for the yield of a reaction and its execution time, the focus could be either on the reaction yield or on the execution time. In the scenario where the reaction yield matters the most, it is related to a higher hierarchy than the execution time. To start the optimization procedure with a lexicographic method, the objectives are sorted in descending order of importance. Each objective is then subsequently optimized without degrading higher-level objectives.^47^ Variants of the lexicographic approach allow for minimal violations of the imposed constraints.^48^,^49^

### Single-objective optimization methods

Most *a priori* methods reformulate multi-objective optimization problems into single-objective optimization problems. The latter are well studied and a plethora of algorithms have been developed for single-objective optimization.^50^–^53^ Some of these algorithms aim to optimize an objective function locally while others aim to locate the global optimum. In some cases, optimization algorithms are based not only on the objective function, but also on its gradients and possibly higher derivatives.

Finding optimal conditions for an experimental setup imposes particular requirements on optimization algorithms as the surface of the experimental objectives is unknown. Additionally, running an experiment can be costly in terms of execution time, money, or other budgeted resources. Therefore, an appropriate optimization algorithm must be gradient-free, and global to keep the number of required objective evaluations to a minimum. In addition, such an algorithm must support optimization on possibly non-convex surfaces. In the following paragraphs we describe four techniques which will be considered herein to study the performance of Chimera.

Systematic grid searches and (fractional) factorial design strategies are popular methods for experimental design.^54^–^56^ These strategies rely on the construction of a grid of parameter points within the parameter (sub-)space, from which points are sampled for evaluation. Grid searches are embarrassingly parallel, as the parameter grid can be constructed prior to running any experiments. However, a constructed grid cannot take into account the most recent experimental results for proposing new parameter points. Moreover, parameter samples proposed from grid searches are correlated, and thus might miss important features of the objective surface or even the Pareto optimal point.

The Covariance Matrix Adaptation Evolution Strategy (CMA-ES) samples parameter points from a multinomial distribution defined on the parameter space.^57^,^58^ After evaluation of all proposed parameter points, distribution parameters are updated *via* a maximum-likelihood approach. As a consequence, the means of the multinomial distribution follow a natural gradient descent while the covariance matrix is updated *via* iterated principal component analysis retaining all principal components. While CMA-ES is successful on highly multi-modal functions, its efficiency drops on well-behaved convex functions.

Recently, Bayesian optimization methods have gained increased attention. Spearmint implements Bayesian optimization based on Gaussian processes.^59^,^60^ Gaussian processes associate every point in the parameter space with a normal distribution to construct an approximation of the unknown objective function. Parameter points can be proposed from this approximation *via* an acquisition function, implicitly balancing the explorative and exploitative behavior of the optimization procedure. While Gaussian process based optimization provides high flexibility, it suffers from the adverse cubical scaling of the approach with the number of observations.

Recently, we introduced Phoenics for a rapid optimization of unknown black-box functions.^61^ Phoenics combines concepts from Bayesian optimization with ideas from Bayesian kernel density estimation. Phoenics was shown to be an effective, flexible optimization algorithm on a wide range of objective functions and allows for an efficient parallelization by proposing parameter points based on different sampling strategies. These strategies are enabled by the introduction of an intuitive bias towards exploitation or exploration.

## Methods

We consider a Pareto optimization problem with *n* objective functions {*f*_*k*_}*n*–1*k*=0 defined on the *d*-dimensional compact subset 
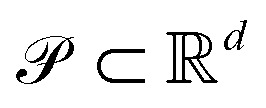
. We further assume that no prior information about the objectives is available and that evaluations of the objectives are demanding in terms of budgeted resources, motivating *a priori* methods with gradient-free global optimization algorithms (see the Background and related work).

In this section, we detail Chimera, which follows the idea of lexicographic methods by providing preference information in the form of a hierarchy in the objectives, but formulates a single ASF based on the provided hierarchy (see Fig. 1). The formulation of the hierarchy in Chimera enables the following procedure: (i) given a hierarchy in the objectives, relative tolerances are defined for each objective, indicating the allowed relative deviation with respect to the full range of objective values. (ii) Improvements on the main objective should always be realized, unless sub-objectives can be improved without degrading the main objective beyond the defined tolerance. (iii) Furthermore, changes in the order of the hierarchy and the tolerances on the objectives should enable the optimization procedure to reach different Pareto-optimal points. Cases where two (or more) objectives are judged to be of equal importance can be accounted for by combining these objectives into a single objective.

### Constructing Chimera

We assume the set of **f** = (*f*_0_,…,*f*_*n*–1_) objective functions to be ordered based on a descending hierarchy, *i.e. f*_0_ is the main objective, and that the optimization procedure aims to minimize each of the objectives. An example of a set of three objective functions is illustrated in Fig. 1A. Chimera is updated at every optimization iteration based on all available observed pairs of parameter points and objectives 
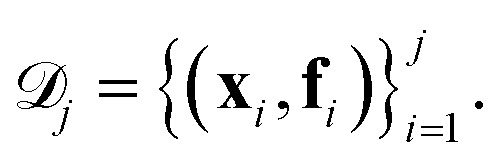
 This provides the additional flexibility to change the order in the importance hierarchy during the optimization process.

Using prior observations 
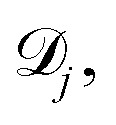
 relative tolerances 
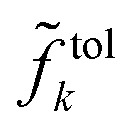
 defined prior to the optimization procedure are used to compute absolute tolerances 
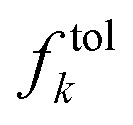
 on all objectives at each optimization iteration (see eqn (1)). Note that absolute tolerances for individual objectives are computed from the minimum and maximum of this objective only in the subset of the parameter space, 
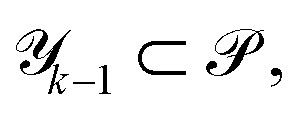
 where the objective one level up the hierarchy satisfies its tolerance criteria (see Fig. 1A).1
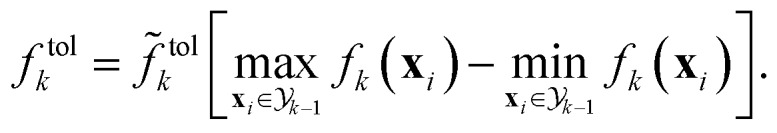



We can determine whether a given objective function value is above or below the given tolerance *via* the Heaviside function *Θ*,2
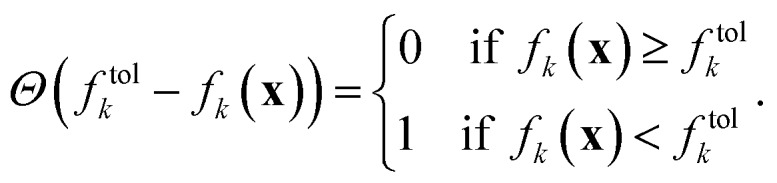



For the following considerations we introduce the abbreviations3
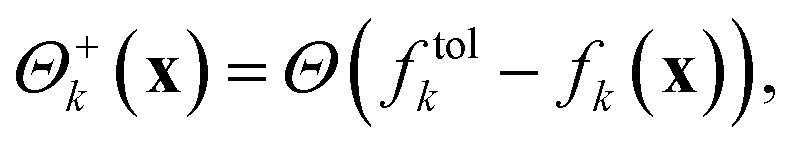

4
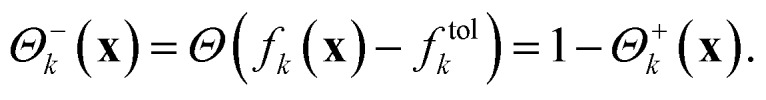



Using the Heaviside function to weight the involved objectives, a single ASF can be constructed. This ASF is sensitive only to a single objective in any region of the parameter space (see Fig. 1A.IV).

However, the assumed values of different objective functions in their respective regions of interest can differ greatly. As such, the value of a lower-level objective might exceed the value of a higher-level objective, as illustrated in Fig. 1A.IV. The decomposition of objectives alone therefore does not present a suitable ASF as parameter regions satisfying tolerances on some objectives might be disfavored due to large values of lower-level objectives. To overcome this limitation we propose to shift objectives *f*_*k*_ based on the minimum of *f*_*k*–1_ in the parameter regions 
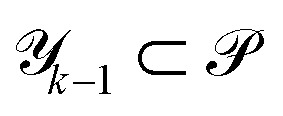
 for which *f*_*k*–1_ does not satisfy the defined tolerance. We denote the shifting parameters with *f*min*k*–1. Chimera *χ*(**x**) is then constructed to account for the hierarchy of individual objectives *via*eqn (5). Following this procedure, the construction and implementation of Chimera are illustrated in Fig. 1.5
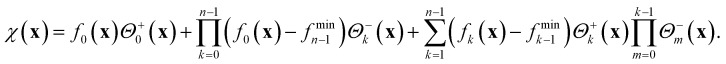



Within this formulation of the ASF, and its associated relative tolerances, a single-objective optimization algorithm is motivated to improve on the main objective. In addition, the algorithm will be encouraged to optimize the sub-objectives as well, from the beginning of the optimization procedure on. Nevertheless, improvements on the sub-objectives will not be realized if they cause degradations in objectives higher up the hierarchy (see Fig. 1B.II). Furthermore, the constructed ASF will be monotonic in proximity to the points in parameter space where Chimera transitions from being sensitive to one objective to being sensitive to another objective if and only if the two objectives do not compete with each other. Detailed explanations on this property of the constructed ASF are provided in the ESI (see Section S.1.3†). Identifying these parameter regions where the ASF is monotonic opens up possibilities for interpretations and the potential discovery of fundamental underpinnings.

As the Heaviside function is not continuous, the constructed ASF also contains discontinuities. However, these discontinuities can be avoided with the logistic function as a smooth alternative to the Heaviside function6
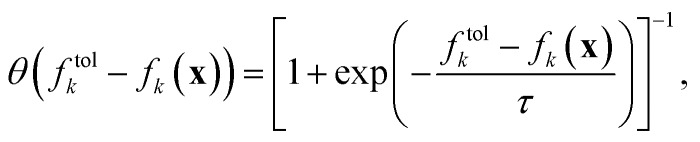
where *τ* > 0 can be interpreted as a smoothing parameter. Note that the logistic function converges to the Heaviside function in the limit 
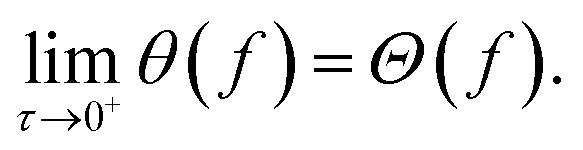
 Fig. 1B depicts Chimera constructed with different values of the smoothing parameter. In general, we observe that small values of *τ* still retain sharp features in the ASF, although discontinuities are lifted. Large values of *τ*, however, may cause a deviation in the global minimum of the ASF and in the location of the Pareto-optimal point.

The impact of the smoothing parameter on the performance of an optimization run is reported in the ESI (see Section S.1.1†). We ran Phoenics on the three one-dimensional objective functions illustrated in Fig. 1 and constructed Chimera with different smoothing parameter values. We find that generally large values of *τ* result in considerable deviations in the objectives after a given number of optimization iterations, eventually causing the optimization algorithm not to find parameter points yielding objectives within the user-defined tolerances. In contrast, small values of *τ* (including *τ* → 0^+^) cause the optimization algorithm to need slightly more objective function evaluations to find parameter points yielding objectives within the defined tolerances. However, we did not observe any significant differences in the performance for intermediate values of *τ*. We recommend the use of *τ* within the [10^–4^, 10^–2^] interval. For all the tests performed and reported in the Results section as well as for the two applications a value of *τ* = 10^–3^ was used.

## Results

The benchmarks presented in this section allow us to assess the ability of Chimera to find Pareto optimal solutions using single-objective optimization algorithms. We start with a focus on the question whether Chimera locates Pareto optimal points for a given set of hierarchies and tolerances. We then proceed with evaluating the performance and behavior of difference single-objective optimization algorithms on Chimera.

To benchmark the performance of Chimera we consider six different sets of well-established analytic objective functions. Five of the sets consist of two objectives, while the sixth set contains three objectives. Details on the objective functions are reported in the ESI (see Section S.1.1†). For all benchmark optimizations reported in this section, we employed the same set of tolerances and constraints on the objectives in the benchmark set, which are reported in the ESI as well (see Section S.1.1†).

### Deviations of the expected optimum from the actual optimum

The performance of Chimera is compared to the behavior of the ASF introduced by Walker *et al.*,^23^ which we will refer to as c-ASF from now on due to its constrained approach. Pareto-optimal points were determined from evaluating each objective on a 1000×1000 grid in the parameter spaces. While tolerances on the objectives for Chimera can be defined *a priori* without detailed knowledge about the shapes of the objectives, the c-ASF introduced requires absolute constraints on the objectives. For a fair comparison between the two ASFs, we therefore also compute constraint values matching the pre-defined tolerances from this grid evaluation.

**Fig. 2:**
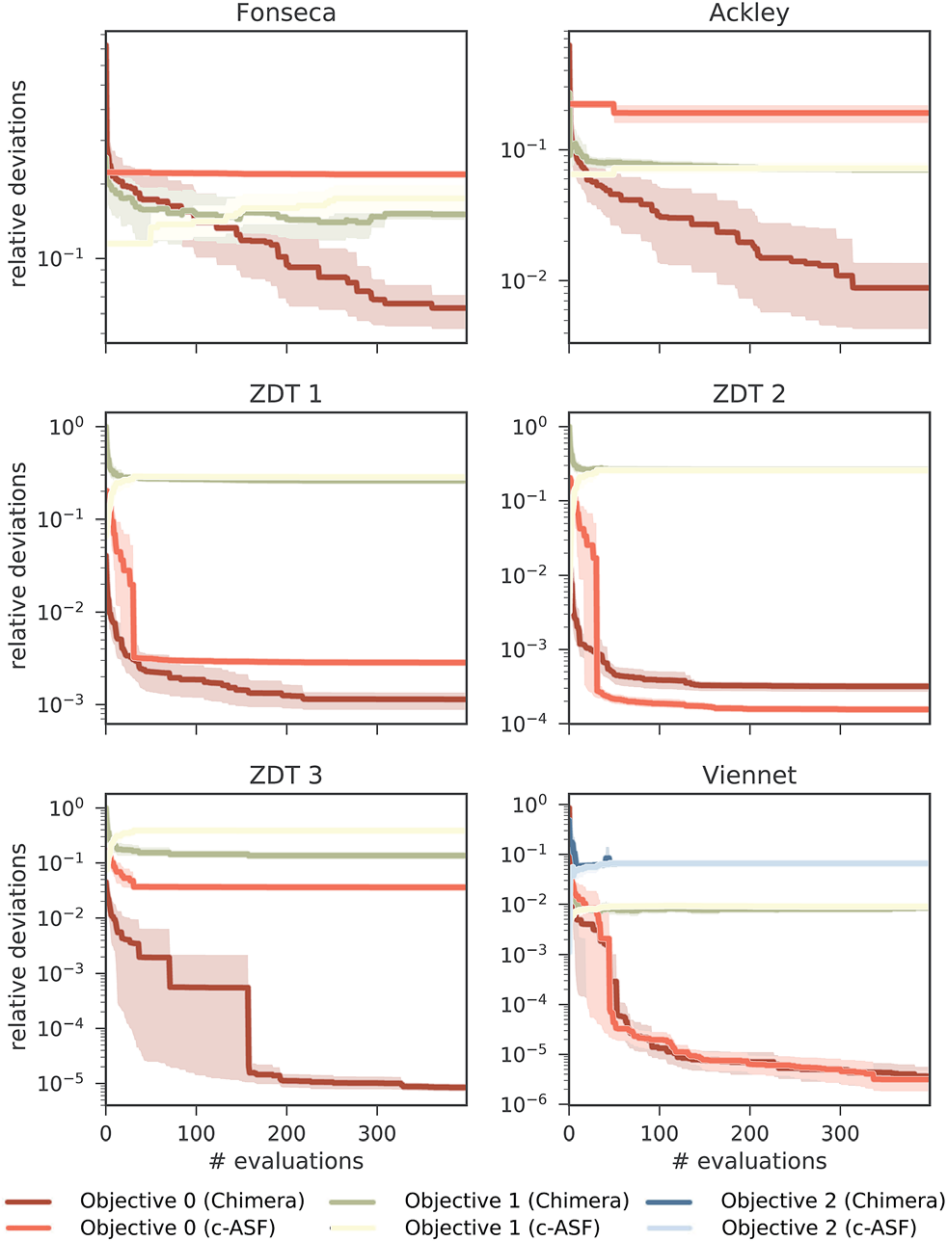
Average relative distance from the Pareto-optimal point determined by the applied constraints. We compare the achieved relative distances of Chimera and c-ASF. Parameter spaces were searched *via* a grid search (see the main text for details).

After these initial computations, we emulate an optimization procedure set up as a grid search, which is a common strategy for experimental design.^54^–^56^ During the optimization procedure we construct both Chimera and c-ASF from the obtained observations. We designed the grid from 20×20 equidistant parameter points. From the resulting 400 grid points, we construct 25 different sampling sequences by shuffling the order of grid points. All objective functions are evaluated at parameter points in sequential order. At each iteration in the optimization procedure, we reconstruct both ASFs and determine their predicted Pareto optimal points. Deviations in the objective values of the predicted Pareto optimal points and the true Pareto optimal points are used as a measure to determine how well Pareto optimal objectives are predicted by either ASF. Average deviations between predicted and true Pareto optimal objectives, with respect to the full range of all objectives, are reported in Fig. 2.

Based on the benchmark results, we find that the Pareto optimal point predicted by Chimera is closer to the true Pareto optimal point with respect to all involved objectives after the full evaluation of the 20×20 grid for four out of the six benchmark sets. With the Viennet benchmark set, we find similar performance in both ASFs, and c-ASF predicts the Pareto optimal point with slightly smaller deviations on the ZDT 2 benchmark set. Details on the benchmark sets are provided in the ESI (see Section S.1.1†).

Besides the prediction accuracy, it is important to emphasize a major difference between Chimera and c-ASF: c-ASF requires detailed knowledge about the individual objective surfaces to set appropriate constraints. The Pareto optimal point can only be determined if reasonable bounds have been defined. In addition, changing the hyperparameters in c-ASF can significantly influence how individual objectives are balanced. Chimera, however, only contains a single hyperparameter *τ* (see eqn (6)), which is used for smoothing the constructed *χ*. From the presented benchmark, we find that Chimera shows good performance with the same choice of *τ* on a diverse set of benchmark functions. We have also illustrated that the performance of an optimization procedure augmented with Chimera only weakly depends on the particular choice of *τ* over several orders of magnitude (see Section S.1.1†).

### Performance with various optimization algorithms

In this section, we report on the performance of four single-objective optimization algorithms on both Chimera and c-ASF. In particular, we employ four gradient-free optimization procedures: grid search,^54^–^56^ CMA-ES,^57^,^58^ spearmint^59^,^60^ and Phoenics.^61^ Details about the optimization procedures are reported in Section S2.2†. The resulting combinations of optimization algorithms and ASFs are then applied to the six analytic benchmark sets, and used to determine how fast the Pareto optimal points can be located.

**Fig. 3:**
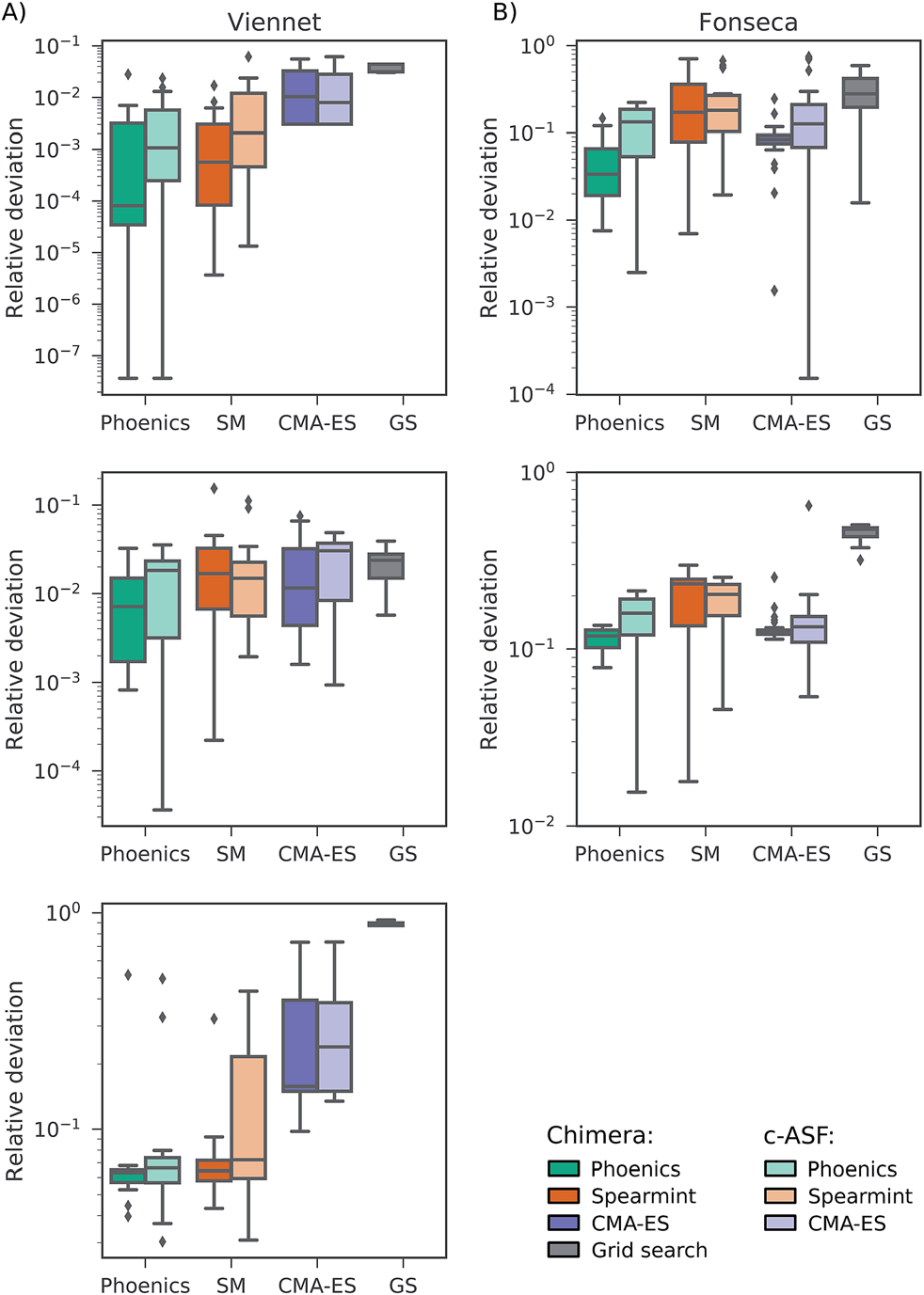
Average smallest relative deviations between objectives sampled by different optimization algorithms after 100 objective function evaluations averaged over 25 different optimization runs. Panel (A) reports results on the Fonseca benchmark set, and panel (B) displays results for the Viennet variant benchmark set.

In all optimization runs we applied the same set of constraints and tolerances as discussed in the previous section. The performance of each optimization algorithm augmented with each of the ASFs is quantified by computing the smallest relative deviation in the objectives between all sampled parameter points and the Pareto optimal point. The average smallest achieved relative deviations after a total of 100 objective set evaluations for the Fonseca set and the Viennet set are reported in Fig. 3. Note that the performance of the grid search does not depend on the ASF, as decisions about which parameter point to evaluate next are not updated based on prior evaluations. Results on the remaining four benchmark sets are reported in the ESI (see Section S.1.4†).

We find that optimization runs of different optimization algorithms augmented with Chimera reach low deviations to the Pareto optimal points after 100 objective set evaluations. When compared to the deviations in objectives achieved by optimization algorithms augmented with c-ASF, Chimera generally seems to lead optimization algorithms closer to the true Pareto optimal objectives. Although the degree of improvement in the deviations of Chimera over c-ASF varies across all objectives, we did not observe a case where c-ASF significantly outperforms Chimera. These observations hold for the duration of the entire optimization procedure, as reflected by the individual optimization traces reported in the ESI (see Section S.1.4†). In particular, the fact that the tolerances are defined relative to the observed range of objectives in Chimera does not appear to be disadvantageous. Indeed, optimization runs with Chimera achieve relatively low deviations in all objectives from the beginning of the optimization procedure on. Furthermore, we find that optimization algorithms based on Bayesian methods (spearmint and Phoenics) generally outperform CMA-ES and grid search, although the degree of improvement can vary with the objectives.

### Behavior of optimization procedures

**Fig. 4:**
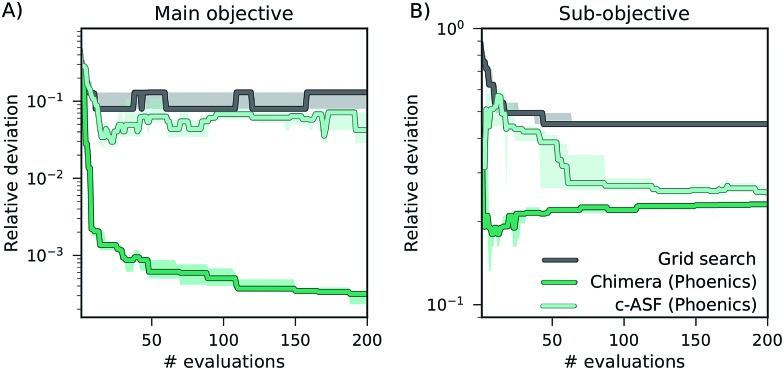
Optimization traces representing the smallest relative deviations between sampled objectives and Pareto optimal objectives averaged over 25 individual optimization runs on the ZDT 2 benchmark set. Panel (A) shows deviations in the main objective, and panel (B) displays deviations in the sub-objective.

In addition to the differences in performance of Chimera and c-ASF with different optimization algorithms, we also observe differences in the general behavior of the optimization runs regarding the trade-off between objectives. The optimization traces generated by optimization algorithms augmented with Chimera closely follow the user-defined hierarchy in the objectives. As such, improvements on sub-objectives are only realized if superior objectives are not degraded beyond the specified tolerance. Optimization runs generated from optimization procedures augmented with c-ASF do not strictly follow this hierarchy. Instead, we observe cases in which c-ASF appears to favor improvements on the sub-objectives even if these improvements cause degradations in superior objectives. An example is given in Fig. 4, where optimization traces of grid search and Phoenics augmented with both ASFs on the ZDT 2 benchmark set are depicted.

While Chimera only allows for improvements on the sub-objective if the main objective is not degraded substantially, c-ASF favors improvements on the sub-objective over improvements on the main objective. This observation, and the fact that this observation can only be made for some of the benchmark sets, corroborates with the functional form of c-ASF. Depending on the considered objectives, improvements on sub-objectives can decrease the penalty term such that degradations in the main objective are allowed. In contrast, Chimera strictly enforces the user-defined hierarchy for a wide range of different objective functions, as demonstrated in this benchmark study.

In summary, the benchmarks presented in this section illustrate that Chimera can identify Pareto optimal points for the provided set of hierarchies and tolerances in the objectives. Moreover, the ASF constructed by Chimera enables a variety of optimization algorithms to locate the Pareto optimal point. Chimera strictly follows the hierarchy imposed by the user and requires less prior information about the shape of the objectives.

Therefore, Chimera is well suited for multi-objective optimization problems where evaluations of the objective functions are costly, satisfying thus the two constraints identified and discussed in the Introduction.

## Applications of Chimera

In this section we demonstrate the applicability and performance of Chimera with two different examples: the auto-calibration of a robotic sampling sequence for direct-injection, and the inverse-design of a four-pigment excitonic system. Both applications involve a larger number of parameters, and include three different objectives to be optimized.

### Auto-calibrating an automated experimentation platform

In this first application we apply Chimera to find optimal parameters for an automated experimental procedure designed for real-time reaction monitoring, as previously reported in the literature.^62^ The procedure is used to characterize chemicals *via* high-performance liquid chromatography (HPLC). The goal of the optimization procedure is to maximize the response of the HPLC, while minimizing the amount of sample used in the analysis along with the overall execution time.

To benchmark the performance of Chimera, experiments were not executed on the robotic hardware, but on a probabilistic model (virtual robot) trained to reproduce the behavior of the real-life experiment. The virtual robot is trained with experimental data collected over two distinct autonomous calibration runs orchestrated by the ChemOS software package.^3^ During this process, both the HPLC response and the execution times were recorded (see the ESI† of ref. 3).

### Constructing a probabilistic model (virtual robot)

The virtual robot was set up as a Bayesian neural network (BNN), which was trained to predict HPLC responses and execution times for any possible set of experimental parameters. These parameters were obtained from 1500 independent experiments conducted fully autonomously, without human interaction.^3^ For these experiments, the six experimental parameters of the procedure were sampled from a uniform distribution, to ensure unbiased and uncorrelated coverage of the parameter space.

For a dense enough sampling of the parameter space, the BNN smoothly interpolates experimental results between two executed experiments. It is important to emphasize that the virtual robot then allows querying experimental results for parameters which have not been realized by the actual experimental setup. As such, the virtual robot trained in this work is well suited to inexpensively benchmark algorithms for experimental design.

The BNN was trained *via* variational expectation-maximization with respect to the network model parameters. Details on the network architecture, the training procedure and the prediction accuracy on both observed (training set) and unobserved data (test set) are reported in the ESI (see Section S.1.5†). The probabilistic model is made available on GitHub.^24^

## Experimental procedure

The goal of this optimization procedure is to (i) maximize the response of the HPLC, (ii) keep the amount of drawn sample low and (iii) minimize the execution time of the experimental procedure. All results presented in this section were obtained with the Phoenics optimization algorithm,^61^ and objectives were sampled from the trained virtual robot. Phoenics was set up with three different sampling strategies, and sequential evaluation of proposed parameter points.

We compare the behavior and performance of Chimera and c-ASF in two different scenarios, defined by different tolerances and constraints on the individual objectives. By sampling the objective space for 10^5^ random uniform parameter points, we can find loose constraints on the objectives such that a parameter point fulfilling all constraints (feasible point) exists. At the same time, such a dense sampling of the parameter space allows us to define a set of objectives which likely cannot be achieved for any set of experimental parameters. As we assume no prior knowledge about the objectives, both scenarios can possibly occur when setting up a new optimization procedure.

Based on the 10^5^ random uniform evaluations of the probabilistic model, we chose the objective constraints reported in Table 1 for both scenarios. Tolerances were defined such that they match up with the constraints relative to the entire range of the observed objective function values. A detailed influence analysis of each parameter on the objectives, as well as the ranges of the observed objectives, is reported in the ESI (see Section S.1.5†).

**Table 1:** Constraints on the objectives for multi-objective optimization runs on the probabilistic model. Uniform sampling of 10^5^ parameter points revealed that loose constraints are achievable by parameter points in a sub-region of the parameter space, while tight constraints cannot be achieved by any parameter point in the parameter space

	Scenario	Response	Sample	Time
Tolerances	Loose	50%	25%	50%
	Tight	20%	10%	10%
Limits	Loose	1250 counts	15 μl	70 s
	Tight	2000 counts	7.5 μl	54 s

### Optimization results

**Fig. 5:**
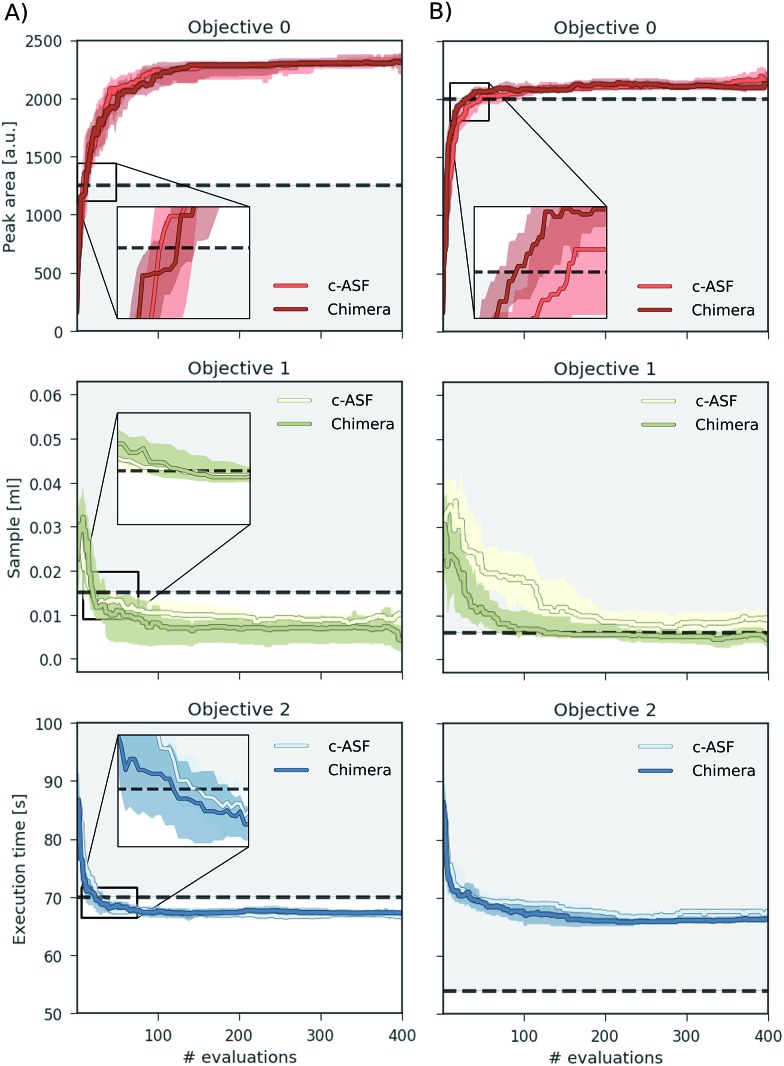
Achieved objective function values for multi-objective optimization runs on a virtual robot model obtained with Phoenics on Chimera and c-ASF averaged over 50 individual runs. The goal of the optimization runs is to maximize the HPLC response, minimize the sample volume and minimize the execution time beyond the set bounds, indicated with black dashed lines.

We carried out a total of 50 optimization runs with different random seeds and a total of 400 optimization iterations for each set of constraints (loose/tight) and each ASF (Chimera/c-ASF). Average traces of the recorded objectives are presented in Fig. 5 for loose constraints (A) and tight constraints (B) as defined in Table 1.

When applying loose constraints to the optimization procedure, we observe a similar behavior of Chimera and the c-ASF. For both cases, Phoenics quickly discovers acceptable HPLC responses above the lower constraint, and is then motivated to further minimize the sample volume and the execution time below the specified bounds. We observe a slight trend of Chimera causing Phoenics to find very large peak areas after conducting more experiments at the advantage of finding still acceptable peak areas at lower solvent amounts earlier on. This trade-off reflects the hierarchical nature of Chimera.

With tight constraints, however, we observe a more significant difference between the two optimization strategies. While with both ASFs Phoenics finds acceptable peak areas much faster than for loose constraints, Chimera appears to help Phoenics in finding acceptable peak areas in fewer experiments. Moreover, the amount of solvent used in the experiments is lower with Chimera from the earliest experiments on, and reaches acceptable levels much faster than with c-ASF. However, the upper bound on the execution time is always exceeded, as there is no point in parameter space for which the peak area is above the chosen lower bound and the execution time below the specified upper bound simultaneously (see Section S.1.5†).

Chimera therefore enables optimization algorithms to rapidly find parameter points yielding objectives close to the user specifications. In the scenario where the parameter point does not exist, Chimera still leads optimization algorithms to parameter points yielding acceptable objective values based on the provided hierarchy and achieves as many objectives as possible.

### Inverse-design of excitonic systems

In this section we demonstrate the applicability of Chimera to inverse-design problems: systems are reverse engineered based on the desired properties. We focus on the design of a system for efficient excitation energy transport (EET). EET phenomena have been of great interest in recent years across different fields such as evolutionary biology or solar cell engineering.^63^–^66^ In particular, studies have focused on understanding the relation between the structure of an excitonic system and its transfer properties fostering the design of novel excitonic devices.

### System definition

The inverse design challenge in this application focuses on an excitonic system consisting of four sites located along the axis **e**_*x*_. Each excitonic site is defined with a position *x*_*i*_ on **e**_*x*_, an excited state energy *ε*_*i*_, a transition dipole with a fixed oscillator strength of |*μ*_*i*_|^2^ = 37.1*D*^2^ and an orientation angle, *φ*_*i*_ = arccos(**e**_*i*_·**e**_*x*_), with respect to the main axis. As such, the excitonic system is fully characterized by a total of ten parameters: four transition dipole orientations, {*φ*_0_, *φ*_1_, *φ*_2_, *φ*_3_}, three relative excited state energies of the last three sites, {*ε*_1_, *ε*_2_, *ε*_3_}, with respect to the excited state energy of the first site *ε*_0_ = 0 and three relative distances between two consecutive sites, {*d*_1_, *d*_2_, *d*_3_}, where *d*_*i*_ = *x*_*i*_ – *x*_*i*–1_ and *d*_0_ = 0. Each of the system parameters was constrained to domains motivated by parameter values for biological light-harvesting complexes.^67^–^70^ Ranges for all parameters are reported in Table 2.

**Table 2:** Parameters for the excitonic system studied in this application. All parameter ranges are inspired by parameter ranges for biological light-harvesting complexes

Parameter	Size	Lower bound	Upper bound
Distances *d*	3	5 Å	40 Å
Energies *ε*	3	–800 cm^–1^	800 cm^–1^
Angles *φ*	4	0	2π

The goal of the optimization procedure is to design excitonic systems with highly efficient energy transport at a low energy gradient across a large distance. These three objectives are quantified as follows: assuming the system transfers excitons from the first site to the fourth site, we compute the total transfer distance as *d* = *d*_1_+ *d*_2_ + *d*_3_. Furthermore, we consider the energy gradient between the first and the last site, *ε* = |*ε*_3_|. Lastly, we also compute the efficiency *η* of the EET. The transfer efficiency is computed from a full population dynamics calculation in the hierarchical equations of motion (HEOM) approach,^71^–^73^ with the QMaster software package, version 0.2.^74^–^77^ HEOM is a numerically exact method which accurately accounts for the reorganization process.

To run a full population dynamics calculation we construct the Frenkel exciton Hamiltonian^78^,^79^ for each proposed excitonic system from the system parameters. The Frenkel exciton Hamiltonian accounts for the excitation energy of each excitonic site and the Coulomb coupling between the sites. While excitation energies are provided as parameters during the optimization, excitonic couplings are computed from the geometry of the system using a point-dipole approximation (see eqn (7)).^80^ We denote the unit vector along the spatial displacement of sites *i* and *j* with **e**_*ij*_ and the distance between the two sites with *d*_*ij*_. Note that the point-dipole approximation only holds for large distances7
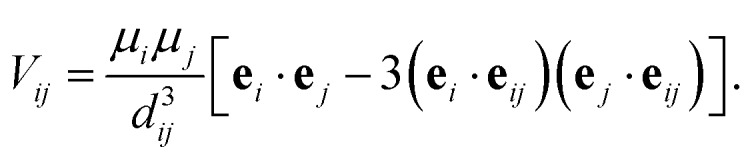



The coupling of the excitonic sites, *J*(*ω*), in the system to the surrounding bath is modeled *via* single-peak Drude–Lorentz spectral densities (see eqn (8)). For all spectral densities, we chose *λ* = 35 cm^–1^ and *v*^–1^ = 50 fs. In all calculations, we use a trapping rate of *Γ*–1trap = 1 ps and exciton lifetimes of *Γ*–1loss = 0.25 ns.8
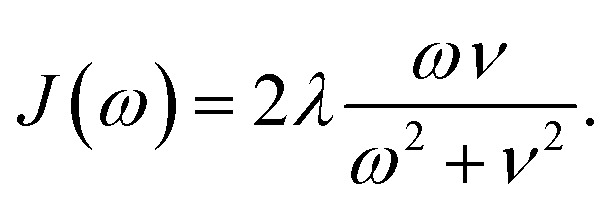



### Optimization procedure

Calculations of the population dynamics on the described excitonic system are computationally demanding, with execution times ranging from about five to about twenty minutes. To accelerate the optimization procedure, we employ Phoenics which allows the generation of multiple excitonic systems per optimization iteration for parallel evaluation. Note that we extended the sampling procedure in Phoenics to account for periodicities in the orientation angles by computing periodic distances when constructing the approximation to the objective function from the kernel density distributions. Details on the procedure are provided in the ESI (see Section S.1.6†).

Phoenics was used with four different sampling strategies, each proposing a different set of parameters in one optimization iteration. For each of the proposed parameter sets, we construct the Frenkel exciton Hamiltonian and start the population dynamics calculation with QMaster. It is important to mention that the execution time of the population dynamics calculation can vary, as it depends on the parameters of the computed system. We therefore set up the optimization procedure in an asynchronous feedback-loop, to process results from population dynamics calculations as soon as they are available. In this feedback-loop, a database is used to store system parameters for future evaluation. When a population dynamics calculation completes, a new set of system parameters obtained from the database is submitted for evaluation. Optimization iterations with Phoenics are triggered right after all three objectives (transfer efficiency, total distance and energy gradient) have been retrieved from the completed population dynamics calculation. At the end of an optimization iteration, the system parameters in the database are updated with the parameters proposed from this optimization iteration.

For the problem of reverse-engineering an excitonic system, we illustrate the performance of Chimera on all possible permutations of hierarchies among all three objectives. For each permutation, we execute a total of 25 individual optimization runs with 400 iterations. All optimization runs aim to design excitonic systems with highly efficient energy transport at a low energy gradient across a large distance. Note that large transfer efficiencies compete with large distances and low energy gradients. To emphasize the importance of large efficiencies and low energy loss of the transport, we chose to apply a tolerance of 10% on the transfer efficiency, 12.5% on the energy gradient and 40% on the total distance.

**Fig. 6:**
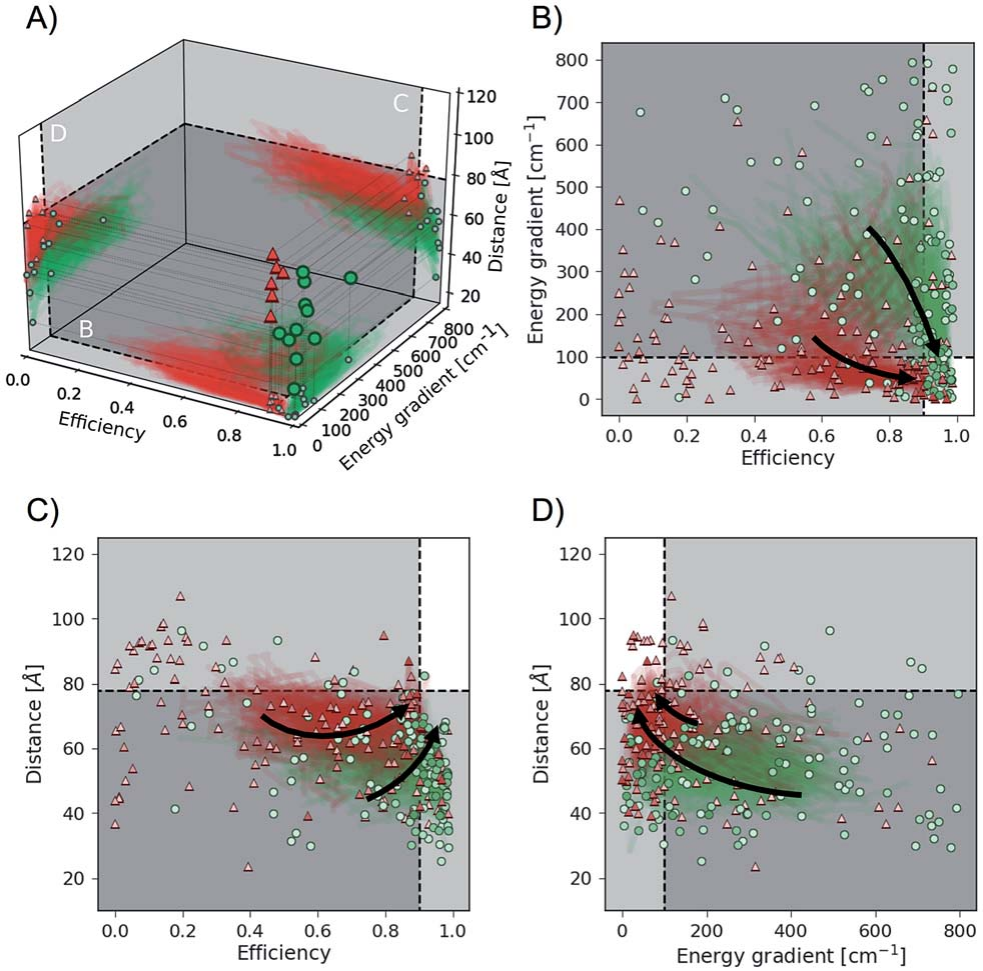
Objective function values sampled in optimization runs with two different hierarchies in the objective. Hierarchy order shown in green dots: (i) transfer efficiency, (ii) energy gradient, (iii) total distance. Hierarchy order shown in red triangles: (i) energy gradient, (ii) transfer efficiency, (iii) total distance. (A) Optimal points with respect to all objectives discovered during individual optimizations. Projections illustrate bootstrapped sampling paths leading to the best performing points. (B–D) Detailed illustration of projected sample traces. Arrows indicate the general paths taken by the optimization algorithm for the different hierarchy orders. More transparent points have been sampled earlier in the optimization procedure, and more opaque points have been sampled at a later stage. White regions indicate the target values for all considered objectives.

We find that Chimera enables Phoenics to discover excitonic systems with the desired objectives in all six studied hierarchy permutations. Details about these permutations are provided in the ESI (see Section S.1.7†). Independently from the order of the objectives in the hierarchy, Chimera guides Phoenics to the parameter space region, for which the associated objectives satisfy all tolerances following different sampling paths. We illustrate this in Fig. 6, which highlights the objectives sampled for two of the six studied permutations: permutation 2 (green dots), which (i) maximizes the transfer efficiency, (ii) minimizes the energy gradient and (iii) maximizes the total distance, and permutation 5 (red triangles) which (i) minimizes the energy gradient, (ii) maximizes the transfer efficiency and (iii) maximizes the total distance. In Fig. 6A we show the points with the most desirable objectives discovered during the optimization runs. Bootstrapped sampling paths leading from the initial (random) points to the best performing points are presented as projections on each of the three planes. Fig. 6b–d further detail the projected paths by supplementing the individually sampled points for each of the permutations.

For both permutations presented in Fig. 6, Chimera successfully leads Phoenics to the region in objective space where all tolerances are satisfied. However, we observe differences in the sampling paths. While with permutation 2 Phoenics samples higher transfer efficiencies earlier on in the optimization procedure, the algorithm is biased towards first sampling lower energy gradients with permutation 5. The sampling paths displayed in Fig. 6 are in agreement with the order of hierarchies in the objectives for the two permutations. These differences in the samplings paths can be rationalized by the fact that high transfer efficiencies and low energy gradients are competing objectives, *i.e.* it is not possible to improve on both objectives with the same changes in the parameters.

Optimization traces for all permutations averaged over the 25 individual optimizations are reported in the ESI (see Section S.1.7†). In accordance with previous results on the analytic benchmarks (see Section S4†) and the auto-calibration of an automated experimentation platform (see Section S5.1†) we find that excitonic systems satisfying the main objective are typically discovered within a few optimization iterations. Sub-objectives are then easily realized in cases where the first and second objectives do not compete, *e.g.* permutation 4, where the first objective is the total distance and the second objective the energy gradient. However, if the first and second objectives do compete with each other (*e.g.* transfer efficiency and energy gradient in Fig. 6) Chimera gradually leads to improvements on the second objective without allowing for degradations in the first objective. This behavior is observed across all studied permutations. Chimera therefore implements the means to realize as many objectives as possible. Based on this observation it can be beneficial to choose the importance hierarchy such that the two most important objectives are expected to not compete with each other in order to accelerate the optimization process.

### Deriving design choices

In the previous sections we observed that optimization algorithms strictly follow the implicit objective hierarchy in the ASF constructed by Chimera. As such, the excitonic systems sampled during the optimization procedure will achieve objectives in the order of the hierarchy imposed. We now study the excitonic systems sampled during the optimization procedures to retrieve design choices made by the algorithm in order to subsequently achieve the objectives in the imposed hierarchy.

**Fig. 7:**
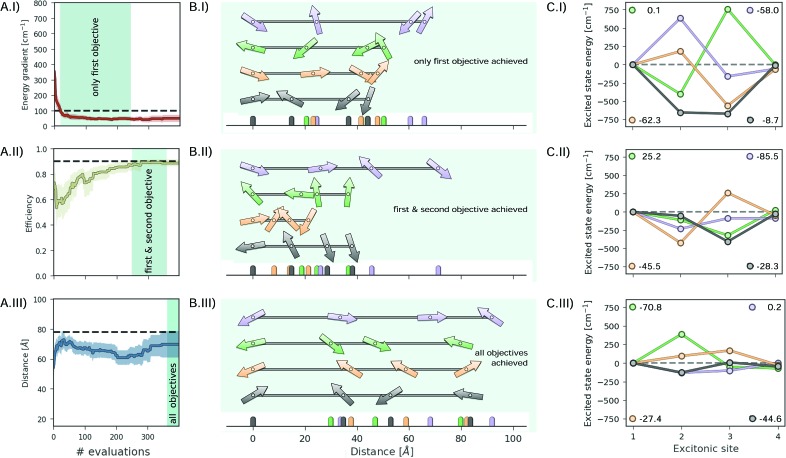
Results for the inverse-design of an excitonic system with (i) a low energy gradient, (ii) high transfer efficiency and (iii) large total distance between the first and the last site. (A) Optimization traces averaged over 25 individual optimization runs, indicating the average required number of designed systems to achieve one, two or all objectives. (B) Illustrations of sampled excitonic systems achieving one, two or three objectives. Arrows represent transition dipoles with their location and orientation to the principal axis. (C) Excited state energies of the systems depicted in (B). The overall energy gradients are reported in the legends.

Fig. 7 illustrates the excitonic systems produced by optimization runs with the following hierarchy: (i) lower the energy gradient, (ii) maximize the transfer efficiency and (iii) increase the total distance covered by the excitonic system. Fig. 7A shows the average optimization traces highlighting the portions where only the first objective is reached, the first and second objectives are reached, and all objectives are reached (Fig. 7A.I–A.III respectively). Since both low energy gradients and large distances compete with high transport efficiency, only a few parameter points satisfy all three objectives.

Fig. 7B illustrates examples of parameters for excitonic systems matching the portions highlighted in Fig. 7A. The depicted excitonic systems are the earliest encountered sets of parameters in these portions. Arrows indicate both the location and the orientation of transition dipoles. Associated excited state energies for these sampled systems are presented in Fig. 7C.

For the sampled excitonic systems achieving the first objective (low energy gradient, Fig. 7I) we do not observe preferences regarding the distances between excitonic sites, orientations of transition dipoles or excited state energies for all but the last sites. These observations are in accordance with the defined objective, as the energy gradient is only controlled by the excited state of the last site.

To subsequently achieve the second objective (high transport efficiency, Fig. 7II) we observe a tendency of sampling shorter overall distances and excited state energies which are lower in magnitude. By further constraining the system to maximize the overall distance (Fig. 7III) transition dipoles are required to align. This sampling behavior provides empirical evidence about the influence of individual system parameters on the considered objectives.

Overall, we find that Chimera is well suited to approach inverse-design challenges and discover systems with desired properties even if the properties of the system are determined by a larger number of parameters. In addition, the formulation of Chimera in terms of a hierarchy in the objectives allows the study of the systems sampled at different stages of the optimization procedure when different objectives are achieved. As demonstrated in the example of designing excitonic systems in Fig. 7, general design choices can be identified empirically from the sampled systems.

## Conclusions

In this work we introduced Chimera, a novel achievement scalarizing function for multi-objective optimization problems associated with experimentation or involved computations. Chimera uses concepts of lexicographic methods to combine any *n* objectives into a single, smooth objective function based on a user-defined hierarchy in the objectives. Additionally, tolerances for acceptable ranges in these objectives can be provided prior to the optimization procedure. Chimera strictly follows the imposed hierarchy in the objectives, and their associated tolerances. This avoids degradation of objectives upon improvement of objectives with lower importance along the hierarchy. Chimera contains a single hyperparameter *τ* controlling the degree of smoothness of the ASF. However, the performance of Chimera appears to be rather insensitive to the value of *τ* across several orders of magnitude. We nonetheless recommend *τ* = 10^–3^ based on our benchmark results. When compared to the formulation of other *a priori* methods, Chimera requires less prior information about the shapes of individual objectives, while providing the flexibility to reach any Pareto optimal point in the Pareto optimal front and keeping the number of objective evaluations to a minimum.

We assessed the performance of Chimera on well-established analytic benchmark sets for multi-objective optimization methods. Our results indicate that Chimera is well suited to predict the location of Pareto optimal points following the provided preference information. Chimera provides additional flexibility by enabling various single-objective optimization algorithms to efficiently run on top of the constructed ASF. In comparison to the general purpose constrained ASF suggested by Walker *et al.*^23^ we find that Chimera enables optimization algorithms to identify Pareto optimal points in fewer objective function evaluations while requiring less detailed knowledge about the objective surfaces.

We further illustrated the capabilities of Chimera for two different applications involving up to ten independent parameters: the auto-calibration of a virtual robotic sampling sequence for direct-injection, and an inverse-design problem for excitonic systems. The auto-calibration application revealed that Chimera always aims to achieve as many objectives as possible following the provided hierarchy and does not improve on sub-objectives if this would imply degradations of the main objective. This observation is also confirmed with the excitonic application. In addition, we found that the imposed hierarchy in the objectives allows the deduction of design principles from sampled parameters. This can find important applications for molecular and structural design with tailored properties. Furthermore, it allows us to understand the influence of distinct features on the global properties of the system.

With the versatile formulation of Chimera, and its low requirements on *a priori* available information, Chimera is readily applicable to problems beyond the scope of the two presented illustrations. We envision Chimera to be successfully used in scenarios where slow merit-evaluation processes such as involved computations or experimentation, most notably in chemistry and materials science, present a challenge to other methods. Moreover, Chimera enables the use of single-objective optimization algorithms and quickly determines conditions yielding the desired merit. As such, Chimera constitutes an important step towards the deployment of self-optimizing reactors and self-driving laboratories, as it provides an approach to overcome the identified constraints: (i) objective evaluations involve timely and costly experimentation, and (ii) no prior knowledge about the objective functions is available.

In summary, we suggest that researchers in automation and more generally multi-objective optimization test and/or employ Chimera for Pareto problems when evaluations of the objectives are expensive and no prior information about the experimental response is available. Chimera is made available on GitHub.^24^

## Conflicts of interest

There are no conflicts to declare.

## Supplementary Material

Supplementary information
