# Supplementary material for: Chimera: enabling hierarchy based multi-objective optimization for self-driving laboratories
Source: Chem Sci. 2018 Aug 28;9(39):7642–55. doi: 10.1039/c8sc02239a (PMC6182568; doi:10.1039/c8sc02239a)
Supplement: Supplementary file 1 [file SC-009-C8SC02239A-s001.pdf]

Electronic Supplementary Material (ESI) for Chemical Science.
This journal is © The Royal Society of Chemistry 2018

Supplementary Information
Chimera: enabling hierarchy based multi-objective optimization for self-driving laboratories

Florian Häse,[1] Loïc M. Roch,[1] and Alán Aspuru-Guzik[2,3,4]

[1]*Department of Chemistry and Chemical Biology, Harvard University, Cambridge, Massachusetts 02138, USA*

[2]*Department of Chemistry and Department of Computer Science, University of Toronto, Toronto, Ontario M5S 3H6, Canada*

[3]*Vector Institute for Artificial Intelligence, Toronto, Ontario M5S 1M1, Canada*

[4]*Canadian Institute for Advanced Research (CIFAR) Senior Fellow, Toronto, Ontario M5S 1M1, Canada**

*Electronic address: alan@aspuru.com

S.1. SUPPLEMENTARY INFORMATION

S.1.1. Benchmark functions

The influence of the smoothing parameter τ on the shape of the achievement scalarizing function has been demonstrated on a set of three one-dimensional objectives, presented in Eq. 1. Note, that all objectives were considered on the $x \in [-1, 5]$. The three objectives are also shown in Fig. 1 in the main text (see Sec. 3.1).

$$f_0(x) = \begin{cases} -2x + 1 & \text{if} \quad x \leq 1 \\ x - 2 & \text{if } 1 < x \leq 3 \\ 7 - 2x & \text{if } 3 < x \leq 3.5 \\ 2x - 7 & \text{if } 3.5 < x \end{cases} \tag{1}$$

$$f_1(x) = 1 - 2\exp[-(x - 2.5)^2] \tag{2}$$

$$f_2(x) = \left((5 - x)^2 + \exp(x - 2)\right)/100 \tag{3}$$

In addition, we benchmarked the performance of Chimera on six well-established analytic sets of objective functions. All of these functions are defined on two dimensional parameter spaces and benchmark sets comprise of two or three different objectives. Contour plots of all objective functions as well as the locations of the global minimum of each objective are presented in Fig. S.3. Note, that for all objective functions the parameter space has always been rescaled to the unit square $[0, 1]^2$ and the objective function values were rescaled to the $[0, 1]$ interval. Python implementations of all objective functions are provided on GitHub.[1]

For all benchmark optimization procedures we chose a particular set of tolerances and limits on the objective functions in each benchmark set. Tolerances and limits used throughout all optimization runs for all benchmark functions are listed in Tab. S.1. Goal of all optimization procedures on the analytic benchmark set is to minimize each individual objective given the defined hierarchies and tolerances.

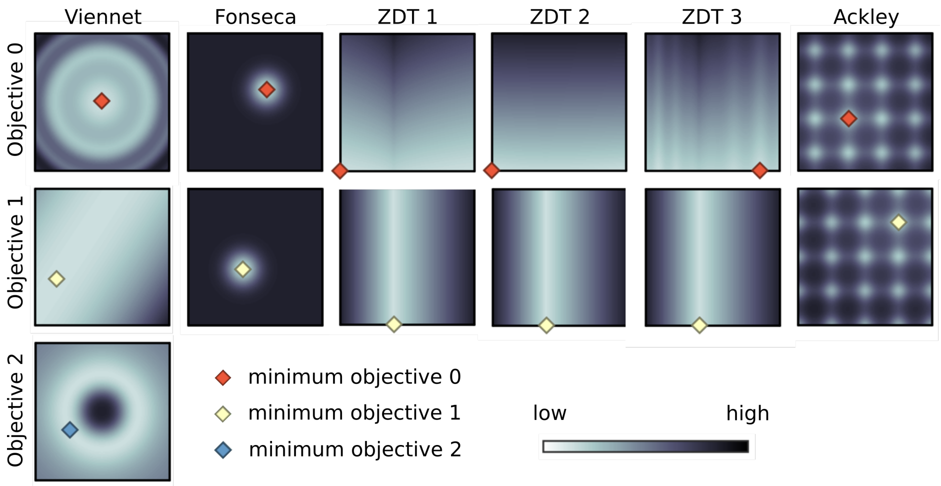

Figure S.1: Contour plots of the employed benchmark functions

Benchmark set	Objective	Tolerance	Limit
Ackley	f_0	50 %	1.808
	f_1	20 %	2.616
Fonseca	f_0	50 %	0.500
	f_1	50 %	0.873
Viennet	f_0	20 %	1.650
	f_1	12 %	17.296
	f_2	20 %	-0.136
ZDT1	f_0	25 %	2.653
	f_1	25 %	0.150
ZDT2	f_0	25 %	2.980
	f_1	25 %	0.150
ZDT3	f_0	25 %	2.391
	f_1	25 %	0.150

Table S.1: Tolerances and limits for all analytic benchmark sets used for assessing the performance of Chimera.

S.1.2. Influence of the smoothing parameter on optimization behavior

In this section we study the influence of the value of the smoothing parameter τ on the behavior of the optimization procedure. On the one hand, smoothing parameters which are too small yield a rather rough ASF, which could be more challenging for the optimization algorithm. On the other hand, smoothing parameters which are too large might smoothen the objectives too much, such that the location of the global optimum of the ASF is shifted away from the Pareto optimal point (see Sec. 3.1). To test this hypothesis, we ran Phoenics on the set of one dimensional objectives presented in Sec. S.1.1 with tolerances of $30\,\%$ on objective 0, $40\,\%$ on objective 1 and $50\,\%$ on objective 2 in combination with different choices of the smoothing parameter τ.

To assess the influence of the value of the smoothing parameter τ on the optimization behavior, we consider an ensemble of 25 individual optimization runs for each smoothing parameter value. For each parameter value, we count how many of the individual optimization runs found parameter points for which all objectives meet the specified tolerances. Results are reported in Fig. S.2 for a total of 400 optimization iterations in each run.

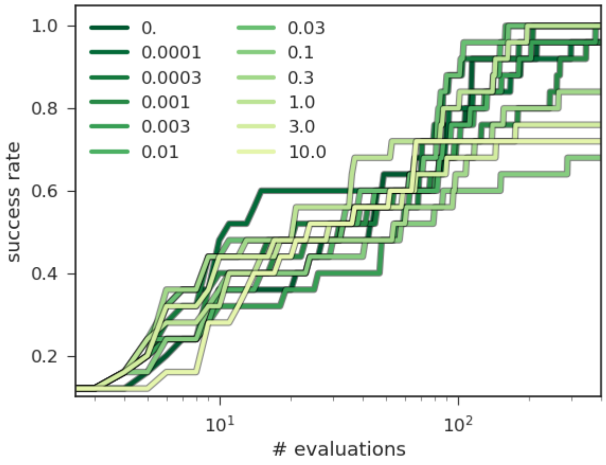

Figure S.2: Rates of successful optimizations on the one dimensional benchmark set out of 25 individual optimization runs conducted with Phoenics on Chimera with different values of the smoothing parameter τ (see legend). Larger values of τ generally show lower success rates later on in the optimization procedures.

In addition to the success rates, we report average optimization traces in Fig. S.3, where we depict the average closest achieved objective function values for all three objectives over the

progress of the optimization. The target values for all three objectives are indicated via dashed lines. Background colors in the plots indicate if the currently best performing parameter set violates objective 0 (red), objective 1 (yellow), objective 2 (blue) or non of the constraints (white).

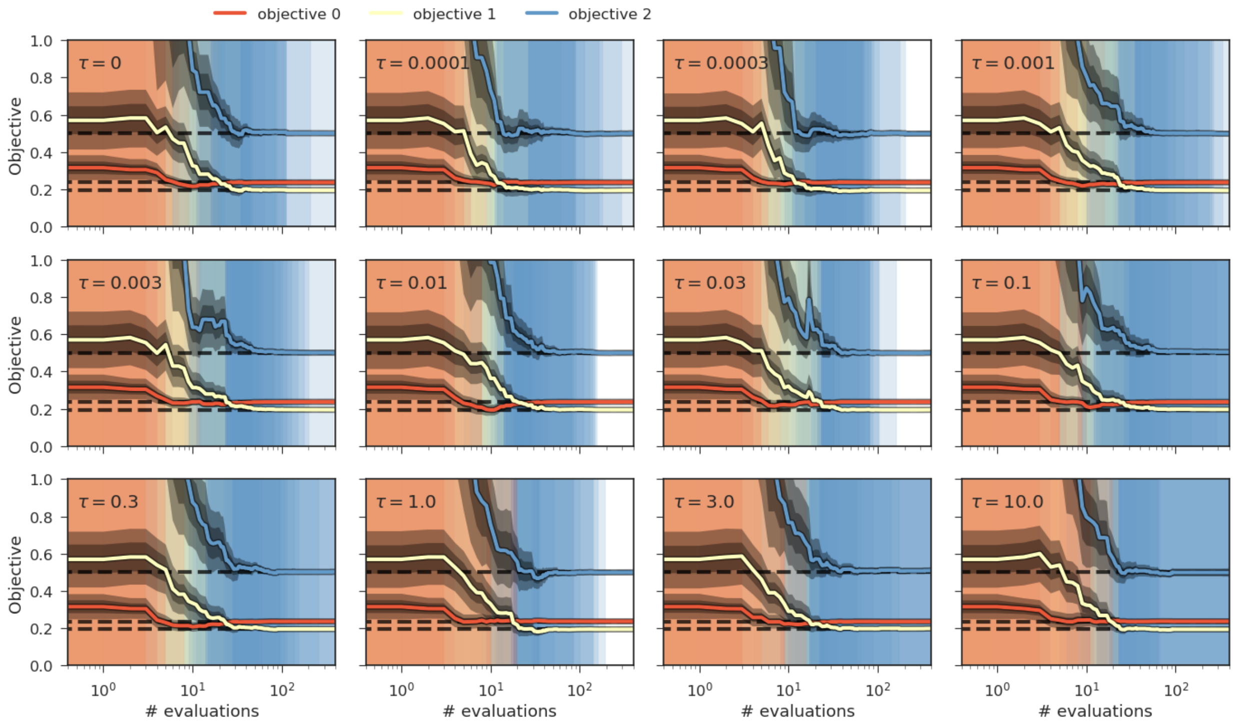

Figure S.3: Influence of the value of the smoothing parameter τ on the convergence of the optimization run. Reported traces have been averaged over 25 individual optimization runs with different random seeds. We do not observe a strong dependence of the optimization behavior on the smoothing parameter.

We observe, that all three objectives are quickly achieved by all optimization runs if the smoothing parameter τ is small ($\tau \leq 0.03$). At the same time, we observe that smoothing parameter values slightly above zero allow for faster convergence towards the target objective values. With these observations we chose to apply a smoothing value of $\tau = 0.001$ to all other optimization procedures presented in this work.

S.1.3. Detecting locally competing objectives

The construction of Chimera allows to locate regions in the parameter space where two particular objectives, f_i and f_j, do not compete with each other locally. Local competition between two objectives can generally be determined via the monotonicity of the two objectives along curves within the considered parameter space region. Note that for smooth f_i and f_j we can always find a finite sized region around any point in the parameter space such that both f_i and f_j are monotonic along any curve within this region. If both f_i and f_j are either monotonically increasing or monotonically decreasing, simultaneous improvements on both objectives are possible and the objectives do not compete. If, however, f_i and f_j differ in their monotonicity then one objective cannot be improved without degrading the other objective. Thus, the two objectives compete with each other.

Chimera is constructed such that it is sensitive only to a single objective in any region of the parameter space, or mostly influenced by a single objective if the Heaviside function is replaced with the logistic function. For two particular objectives, f_i and f_j, we refer to $\mathcal{R}_i$ as the region where Chimera is mostly sensitive to f_i, and to $\mathcal{R}_j$ the region where Chimera is mostly sensitive to f_j. Furthermore, we denote with $\{x^*\}$ the set of points transitioning from $\mathcal{R}_i$ to $\mathcal{R}_j$. Without loss of generality we assume a hierarchy where f_i is assigned a higher importance than f_j.

Within this definition, the two objectives f_i and f_j do not compete with each other locally in finite sized regions around parameter points $\{x^*\}$ if and only if Chimera is monotonic within these regions.

First, we consider the implication of Chimera being monotonic if f_i and f_j do not compete with each other. If f_i and f_j do not compete, both are either monotonically increasing or monotonically decreasing along every curve passing through x^*. By construction, the values of Chimera are larger for parameter points in $\mathcal{R}_i$ than for parameter points in $\mathcal{R}_j$ as f_i is assigned the higher importance. The transition occurs because f_i assumes low values close to the transition point x^* and reaches its tolerance criterion (see main text Fig. 1). Thus, we can find a region within $\mathcal{R}_i$ where f_i monotonically increases along a curve directed towards x^*. Based on our assumption, f_j is monotonically decreasing along the same curve. Therefore, Chimera is also monotonic along

this curve.

Next, we consider the implication of f_i and f_j not competing with each other in proximity to a transition point x^* if Chimera is monotonic along curves passing through x^*. Again, Chimera assumes larger values in $\mathcal{R}_i$ than in $\mathcal{R}_j$ by construction. We define a curve going from $\mathcal{R}_i$ to $\mathcal{R}_j$ while passing through x^*. By assumption, Chimera is monotonically decreasing along this curve. Since Chimera is dominated by f_i in $\mathcal{R}_i$ and dominated by f_j in $\mathcal{R}_j$, both f_i and f_j are monotonically decreasing along this curve and thus do not compete.

This behavior of Chimera can be exploited when analyzing relations between different objectives. In particular, this behavior provides a qualitative tool to identify locally competing or non-competing objectives. Analyzing the parameter region in which objectives are locally competing might reveal insights into fundamental underpinnings of the competition.

S.1.4. Analytic benchmarks

We benchmarked Chimera by running a number of optimization algorithms based on different methods on six well-established analytic benchmark sets introduced in Sec. S.1.1. Details of the benchmark studies are provided in the main text (see Sec. 4.2). In this section, we report the complete results of this benchmark study.

S.1.4.1. Performance after completion of the optimization procedures

In Fig. S.4, we report the average smallest achieved relative deviation of objectives from Pareto optimal objectives for the remaining four benchmark sets: ZDT1, ZDT2, ZDT3 and Ackley. Benchmark results on the Fonseca and the Viennet sets are reported in the main text (see Sec. 4.2). Four different optimization algorithms (grid search, CMA-ES, spearmint, and Phoenics; see Sec. 2 for details) have been run on both Chimera and c-ASF for a total of 100 optimization iterations.

In accordance with results on the Fonseca and the Viennet variant benchmark sets (see Sec. 4.2)

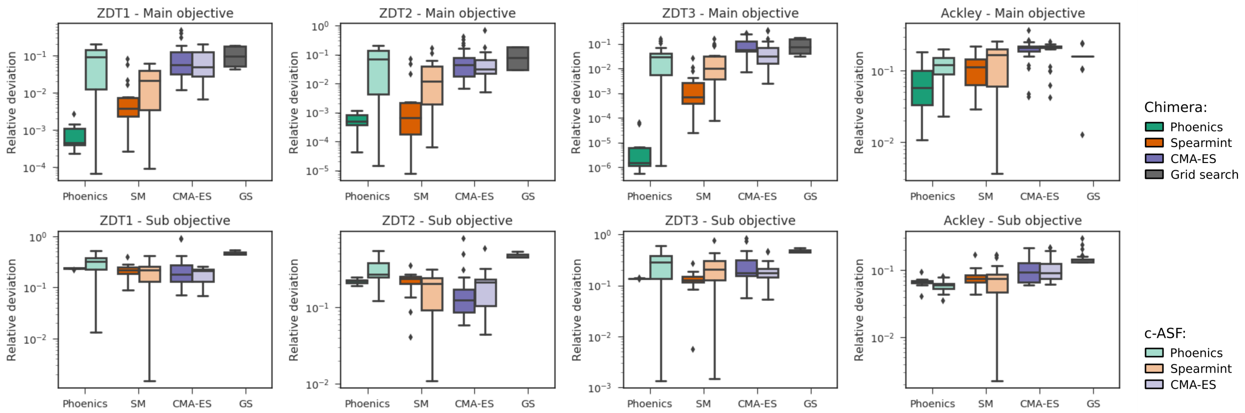

Figure S.4: Average smallest deviations from Pareto optimal points achieved by the four studied optimization algorithms on the remaining benchmark sets. Results are averaged over 25 independent runs executed for 100 iterations each.

we find that Chimera leads the optimization algorithms closer to the Pareto optimal values with the same number of function evaluations.

S.1.4.2. Performance during the optimization procedures

In addition to the average achieved smallest deviations at the end of the optimization procedures we also report the traces of achieved objectives over the duration of the optimization procedure. Fig. S.5 depicts the objective traces for all six benchmark functions after every iteration of the optimization procedures. Uncertainty bands highlight the 68 % confidence interval computed from the 25 repetitions of each optimization run.

Overall, we observe that Chimera enables the studied optimization algorithms to get closer to the Pareto optimal values faster from the very beginning of the optimization procedure. The fact, that tolerances are defined with respect to the current observed minima and maxima of the objectives therefore does not seem to significantly delay the optimization procedure.

Furthermore, as reported in the main text (see Sec. 4.2), we find that Chimera samples points in accordance with the imposed hierarchy, i.e. improvements on the sub-objective are not realized if they come along with degradations on the main objective. However, c-ASF sometimes shows this behavior.

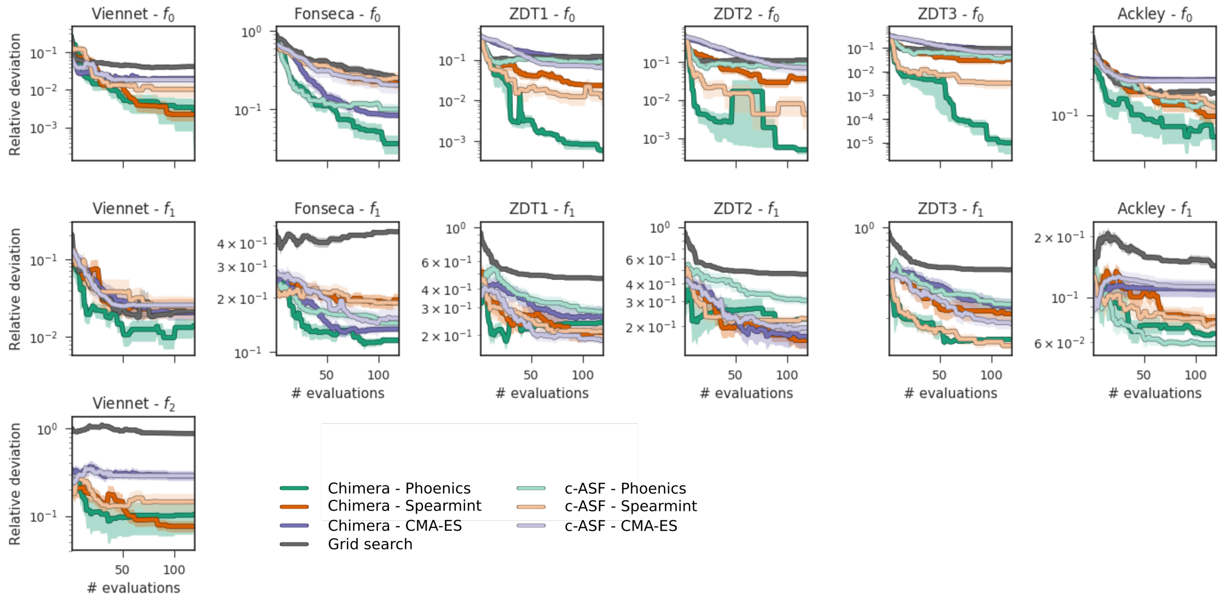

Figure S.5: Optimization traces depicting the smallest deviations between sampled objectives and Pareto optimal objectives averaged over 25 optimization runs with the four employed optimization algorithms on Chimera and c-ASF. Uncertainty bands highlight the 68 % confidence interval computed from the 25 repetitions of each optimization run.

S.1.5. Virtual model of the N9

In this section we detail the construction of a probabilistic model to reproduce and interpolate experimental results obtained from autonomous calibrations of a robotic sampling sequence for direct-inject HPLC analysis.[2] Provided sufficient coverage of the space of experimental parameters, the trained probabilistic model can then be used to query experimental results for any set of experimental conditions without running the experiment.

S.1.5.1. Dataset of experimental results

The experimental procedure consists in the characterization of an unknown chemical sample via high-performance liquid chromatography (HPLC). The calibration of the setup involves an optimization of six experimental parameters. For each experiment, the response of the HPLC as well as the execution time of the experiment can be measured. Details of the experimental procedure are described elsewhere.[2]

An autonomous sampling sequence of the experimental procedure allowed to the efficient execution of a total of $1,500$ individual experiments for the acquisition of experimental outcomes for given experimental conditions. Parameters for the experimental procedure were generated by uniformly sampling the parameter space, to ensure uniform and uncorrelated coverage of the parameter space. Experimental results are depicted in Fig. S.6

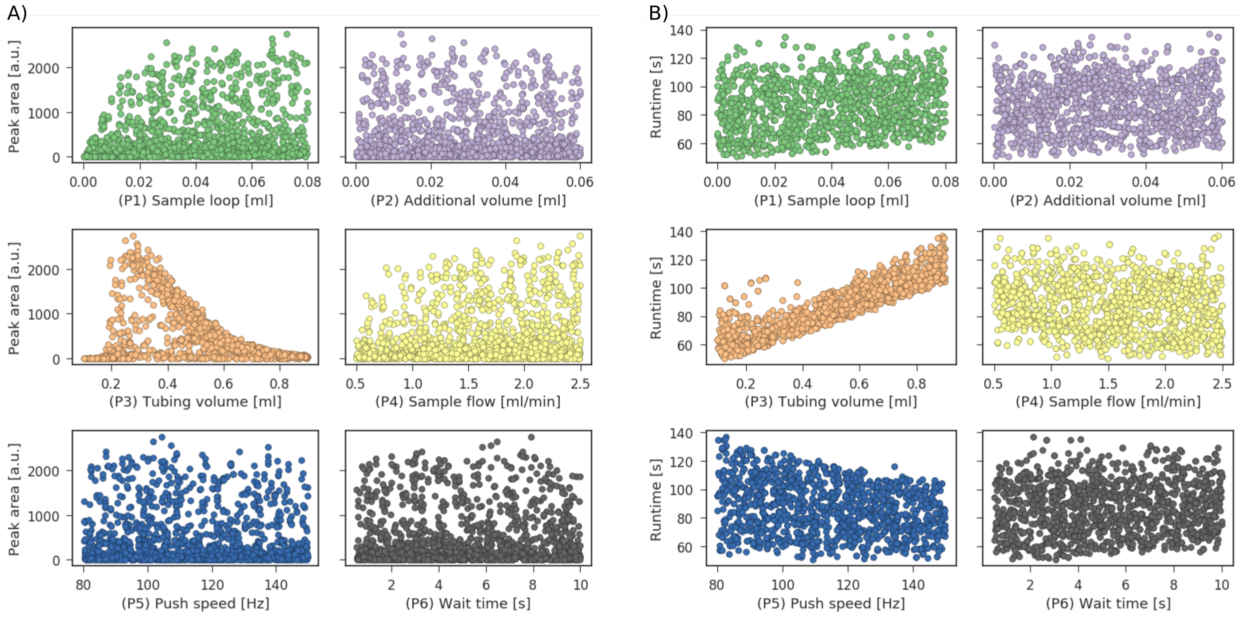

Figure S.6: Experimental results of individual auto-calibration experiments on the robotic sampling sequence. Panel (A): Achieved peak areas for different parameter choices. Panel (B): Achieved execution times for different parameter choices.

S.1.5.2. Training a Bayesian neural network

We construct a test set by randomly sampling 10 % of all points in the dataset. From the remaining 90 % of the dataset, we select the most diverse 80 % for the training set based on principal component analysis (PCA) analysis following a procedure reported in the literature.[3] The remaining 10 % of the dataset are used as a validation set for early stopping.

The probabilistic model was set up as a fully connected Bayesian neural network with three

layers and 192 neurons per layer. Distributions of weights and biases were adapted via variational expectation-maximization, which was carried in Edward, version 1.3.5,[4] with the Adam optimization algorithm,[5] a learning rate of $10^{-2.5}$, and 100 randomly chosen training points per batch.

For the peak area, one of the experimental results to reproduce, we observe that some of the experimentally obtained values are exactly zero, but never negative. To incorporate these features in our model, we employ a modified version of the leaky ReLU activation function, as shown in Eq. 4. The modification to the traditional leaky ReLU consists in splitting the activation function into three piece-wise linear parts. While positive inputs $x > 0$ are processed just like in traditional ReLU or leaky ReLU activation functions, negative inputs are mapped onto zero if the inputs are slightly negative, but mapped onto a linear function with small slope for large negative inputs.

$$f(x) = \begin{cases} x & \text{if } 0 < x \\ 0 & \text{if } -dx < x \leq 0 \\ \alpha x & \text{if } x \leq -dx \end{cases} \tag{4}$$

We chose $\alpha = 0.1$ and $dx = 2$. With this choice of the leakage parameter, the activation function is flat for inputs $x \in [-2, 0]$. Weights and biases were regularized via a Laplacian prior, corresponding to L1 regularization in traditional neural networks. Every 200 training epochs we computed the prediction accuracy on the training and the validation set by sampling predictions from 200 network instances. Network training was aborted if the prediction error on the validation set was found to either increase or to be twice as large as the prediction error on the training set.

Fig. S.7 illustrates the prediction accuracies on the peak areas and execution times obtained by averaging over 200 samples after completion of the training procedure. Prediction errors for the two experimental results are reported in Tab. S.2. Correlations between predicted properties and target properties of 97.8 % f for the peak areas and 99.7 % for the execution times.

Dataset	HPLC response [a.u.]	Execution time [s]
Training	117.16	2.20
Validation	147.04	1.90
Test	193.64	1.75

Table S.2: RMSDs of experimental results predicted by the trained Bayesian neural network from the actual experimental results for all three datasets.

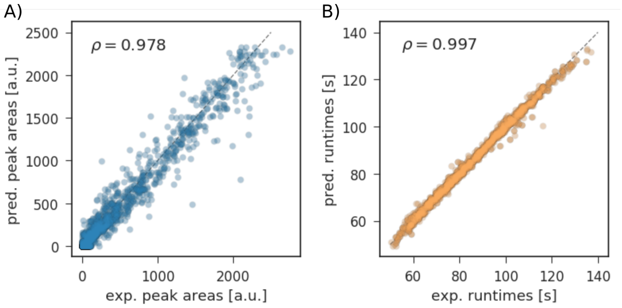

Figure S.7: Predictions of Bayesian neural networks compared to experimental results on the N9 system. Panel (A) displays scatter plots for comparing peak areas, and panel (B) shows results for execution times. The lines of perfect agreement are represented in black. Pearson correlation coefficients ρ are reported for both properties.

S.1.6. Optimizations on periodic domains

The inverse-design problem of finding excitation energy transfer systems discussed in the main text (see Sec. 5.2) involves a total of ten independent parameters. Four of these parameters describe the orientation of transition dipoles with respect to a principal axis, expressed in terms of an angle $\varphi \in [0, 2\pi]$. The orientation of the transition dipoles is periodic, which imposes a constraint on the response surface of objectives with respect to these parameters. This constraint can be taken into account when constructing approximations to response surfaces during the optimization procedure. Indeed, by accounting for this periodicity constraint, a more accurate approximation to the response surface can be found, which has the potential to determine the location of the global minimum in fewer optimization iterations.

In this section, we demonstrate how Phoenics can be expanded to account for periodic

boundary conditions on the parameter domain. Phoenics constructs approximations to an objective function by estimating the kernel density of observed parameter points and reweighting those by the corresponding observed objective function value.[6] Kernel densities p_k are estimated via a Bayesian neural network as shown in Eq. 5. The Bayesian neural network is used to sample random variables ϕ_3 in the parameter domain based on previously observed parameter points $\boldsymbol{x}_k$. Ref. [6] provides a detailed description of the construction of kernel densities

$$p_k(\boldsymbol{x}) = \left\langle \sqrt{\frac{\tau_n}{2\pi}} \exp\left[-\frac{\tau_n}{2} \left(\boldsymbol{x} - \phi_3(\boldsymbol{\theta}; \boldsymbol{x}_k) \right)^2 \right] \right\rangle_{\text{BNN}} . \tag{5}$$

Importantly, the construction of the kernel densities p_k at an arbitrary point $\boldsymbol{x} \in \mathbb{R}^d$ in the parameter domain depends on the distance $d(\boldsymbol{x}, \phi_3(\boldsymbol{\theta}; \boldsymbol{x}_k)) = \boldsymbol{x} - \phi_3(\boldsymbol{\theta}; \boldsymbol{x}_k)$ between this parameter point $\boldsymbol{x}$ and the random variable $\phi_3(\boldsymbol{\theta}; \boldsymbol{x}_k)$ sampled from the Bayesian neural network. We now consider a scenario where the objective f is periodic with periodicity $\boldsymbol{P}$, i.e. $f(\boldsymbol{x}) = f(\boldsymbol{x} + \boldsymbol{P})$ for all $\boldsymbol{x} \in \mathbb{R}^d$. A periodicity constraint on the parameter domain can be formulated by replacing this distance $d(\boldsymbol{x}, \phi_3(\boldsymbol{\theta}; \boldsymbol{x}_k))$ by a periodic distance $d_{\text{periodic}}(\boldsymbol{x}, \phi_3(\boldsymbol{\theta}; \boldsymbol{x}_k))$.

Computing the periodic distance from all periodic images of the kernel density is computationally costly. As a compromise between the computational demand of the approach and accuracy of the periodicity constraint, we only account for nearest periodic images and neglect higher order periodic images. This approximation becomes more accurate with more optimization iterations, as the precision τ_n increases.

We illustrate the construction of periodic objective function approximations one a one-dimensional example. The considered objective function f consists of the product of two cosine functions, as shown in Eq. 6, with a period of $P = 1$. Phoenics was used to determine the location of the global minimum of this function within the $x \in [0, 1]$ interval by constructing the approximation with and without periodicity support. Note, that the global minimum is located at $x^* = 0.05$.

$$f(x) = -\cos\left((\pi(x - 0.05)) \cos\left(3\pi(x - 0.05) \right) \right) \tag{6}$$

Fig. S.8 shows the approximations constructed to the objective function after two, five and eight optimization iterations with and without periodicity support. We find that the optimization run without periodicity support tend to sample the objective function at large values of x. Only after a few optimization iterations, the location of the global minimum at small values of x is discovered. In contrast, the optimization procedure supporting periodicity in the objective function discovers the location of the global minimum within much fewer iterations, and needs fewer observations to construct reasonable approximations to the objective function.

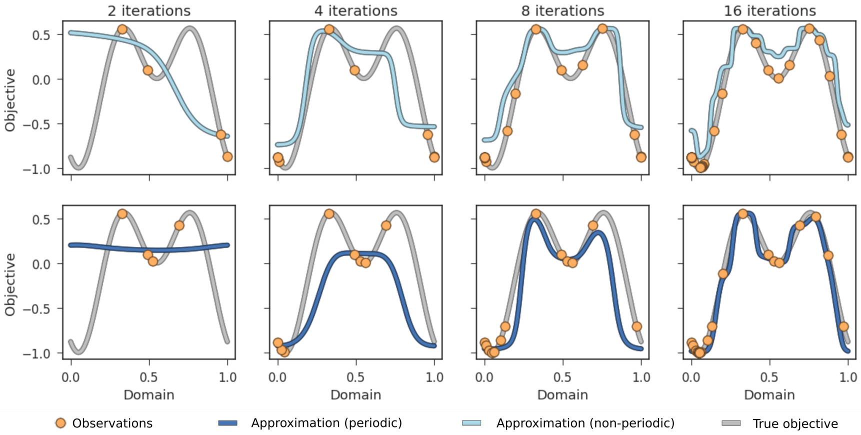

Figure S.8: Optimization runs using Phoenics on a periodic one-dimensional objective function. Upper panels depict the approximation to the objective function constructed by Phoenics without accounting for the periodicity of the objective. Lower panels, in contrast, depict the approximations constructed with periodicity taken into account.

S.1.7. Excitonics application

Here we present the average optimization traces for all studied objective hierarchy permutations of the excitonics application. While the order of the hierarchy in the objectives was changed between different permutation runs, all other parameters such as tolerances were kept the same. Fig. S.9 shows optimization traces for all six permutations of hierarchies averaged over 25 individual optimization runs. Optimization traces are sorted by hierarchy from top to bottom for all permutations.

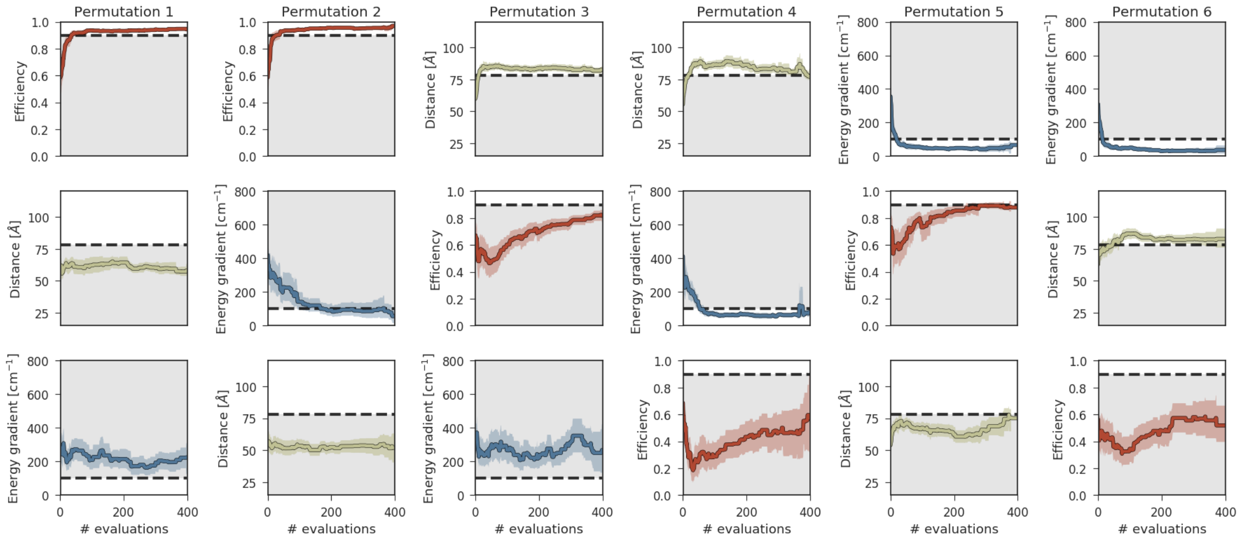

Figure S.9: Optimization traces for the three objectives in the excitonics application averaged over 25 individual optimization runs for all six possible permutations. Top panels present the optimization traces for the main objectives in each permutation, central panels the optimization traces for the sub-objective and bottom panels the traces for the least important objective. Dashed black lines indicate the lower/upper limits on each of the objectives.

---

[1] F. Häse, L. M. Roch, C. Kreisbeck, and A. Aspuru-Guzik, GitHub (2018), URL `https://github.com/aspuru-guzik-group/phoenics`.

[2] L. M. Roch, F. Häse, C. Kreisbeck, T. Tamayo-Mendoza, L. P. E. Yunker, J. E. Hein, and A. Aspuru-Guzik, chemRxiv preprint chemRxiv:5953606 (2018).

[3] F. Häse, C. Kreisbeck, and A. Aspuru-Guzik, Chem. Sci. **8**, 8419 (2017).

[4] D. Tran, A. Kucukelbir, A. B. Dieng, M. Rudolph, D. Liang, and D. M. Blei, arXiv preprint arXiv:1610.09787 (2016).

[5] D. P. Kingma and J. Ba, arXiv preprint arXiv:1412.6980 (2014).

[6] F. Häse, L. M. Roch, C. Kreisbeck, and A. Aspuru-Guzik, arXiv preprint arXiv:1801.01469 (2018).
